# Unique Evolution of the UPR Pathway with a Novel bZIP Transcription Factor, Hxl1, for Controlling Pathogenicity of *Cryptococcus neoformans*

**DOI:** 10.1371/journal.ppat.1002177

**Published:** 2011-08-11

**Authors:** Seon Ah Cheon, Kwang-Woo Jung, Ying-Lien Chen, Joseph Heitman, Yong-Sun Bahn, Hyun Ah Kang

**Affiliations:** 1 Department of Life Science, Research Center for Biomolecules and Biosystems, College of Natural Science, Chung-Ang University, Seoul, Korea; 2 Center for Fungal Pathogenesis, Seoul National University, Seoul, Korea; 3 Department of Biotechnology, Center for Fungal Pathogenesis, Yonsei University, Seoul, Korea; 4 Department of Molecular Genetics and Microbiology, Medicine, and Pharmacology and Cancer Biology, Duke University Medical Center, Durham, North Carolina, United States of America; Washington University School of Medicine, United States of America

## Abstract

In eukaryotic cells, the unfolded protein response (UPR) pathway plays a crucial role in cellular homeostasis of the endoplasmic reticulum (ER) during exposure to diverse environmental conditions that cause ER stress. Here we report that the human fungal pathogen *Cryptococcus neoformans* has evolved a unique UPR pathway composed of an evolutionarily conserved Ire1 protein kinase and a novel bZIP transcription factor encoded by *HXL1* (*HAC1* and *XBP1*-*L*ike gene *1*). *C. neoformans HXL1* encodes a protein lacking sequence homology to any known fungal or mammalian Hac1/Xbp1 protein yet undergoes the UPR-induced unconventional splicing in an Ire1-dependent manner upon exposure to various stresses. The structural organization of *HXL1* and its unconventional splicing is widely conserved in *C. neoformans* strains of divergent serotypes. Notably, both *C. neoformans ire1* and *hxl1* mutants exhibited extreme growth defects at 37°C and hypersensitivity to ER stress and cell wall destabilization. All of the growth defects of the *ire1* mutant were suppressed by the spliced active form of Hxl1, supporting that *HXL1* mRNA is a downstream target of Ire1. Interestingly, however, the *ire1* and *hxl1* mutants showed differences in thermosensitivity, expression patterns for a subset of genes, and capsule synthesis, indicating that Ire1 has both Hxl1-dependent and -independent functions in *C. neoformans*. Finally, Ire1 and Hxl1 were shown to be critical for virulence of *C. neoformans*, suggesting UPR signaling as a novel antifungal therapeutic target.

## Author Summary

The unfolded protein response (UPR) is a widely conserved signaling pathway in eukaryotic cells and protects cells from the ER stress causing accumulation of toxic unfolded or misfolded proteins. Nevertheless, the UPR pathway has been poorly exploited as a therapeutic target for treatment of eukaryotic fungal pathogens, mainly due to its evolutionarily conserved features. The present study reports unique evolution of the UPR pathway in the basidiomycetous human fungal pathogen, *Cryptococcus neoformans*, which causes life-threatening meningoencephalitis in both immunocompromised and immunocompetent individuals. Here, for the first time we identified and characterized the *C. neoformans* UPR pathway, which is composed of an evolutionarily conserved and a distinct signaling component, an ER stress sensor Ire1 and its downstream bZIP transcription factor Hxl1, respectively. Intriguingly, *Cryptococcus* Hxl1 is very divergent from yeast Hac1 and human Xbp1, but subject to Ire1-mediated unconventional splicing. The Ire1-Hxl1-dependent UPR pathway functions not only in the major response to ER stress, but also plays critical roles in controlling cell wall integrity, growth at host physiological temperature, antifungal drug susceptibility, and virulence of *C. neoformans*. Therefore we propose Hxl1 is an ideal target for antifungal drug development, based on its marked divergence from the host Xbp1 transcription factor.

## Introduction

In eukaryotic cells, the endoplasmic reticulum (ER) is the site where most secreted and plasma membrane proteins are synthesized, folded, and matured. Impairment of protein folding results in accumulation of toxic unfolded or misfolded proteins, which causes ER stress and instigates intracellular signaling from the ER to the nucleus. ER stress is also induced by altered calcium homeostasis and glycosylation, nutrient starvation, pathogen infection, and oxidative stress [1]. Mounting evidence links ER stress to human diseases, as diverse as diabetes, viral infection, Alzheimer, cancer, and inflammation [2], [3]. To alleviate the ER stress, eukaryotic cells activate a conserved unfolded protein response (UPR) signaling pathway, which regulates the expression of numerous genes encoding ER chaperones and folding enzymes as well as proteins involved in diverse cellular metabolisms [1], [4].

In the budding yeast *Saccharomyces cerevisiae*, an unfolded protein sensor, Ire1 and its sole downstream transcription factor, Hac1, were discovered and characterized as the key signal components that mediate the UPR pathway [5]. As a type I transmembrane Ser/Thr kinase with an endoribonuclease (RNase) domain, Ire1 senses ER stress in the ER lumen and undergoes activating autophosphorylation. Subsequently activated Ire1 removes an unconventional intron from the *HAC1* mRNA, which encodes a basic domain/leucine zipper (bZIP) transcription factor [6]. The unspliced *HAC1* mRNA is not translated due to long-range base pairing interactions between the *HAC1* 5′-untranslated region and its intron. Therefore, this Ire1p-dependent unconventional splicing of the *HAC1* mRNA is a critical and unique regulatory process for activation of UPR target genes [7]. Similar to yeast, upon activation of the UPR pathway, mammalian *XBP1* mRNA encoding an ortholog of yeast Hac1 is spliced unconventionally by the RNase activity of Ire1α. The activated Xbp1 transcription factor is subsequently translated from the spliced *XBP1* mRNA [1]. Unlike yeast Hac1, however, the unspliced *XBP1* mRNA is translated, generating a negative regulator of the UPR pathway [8]. In mammalian cells, besides Xbp1, UPR is also governed by two ER-membrane-associated bZIP transcription factors, ATF6, and PERK [9]. ATF6 is a type II transmembrane protein normally retained in the ER but transported to the Golgi, where it is subjected to proteolytic process. The released N-terminal fragment of ATF6 containing bZIP DNA binding domain and transcriptional activation domains is translocated to the nucleus where it activates the transcription of ER stress response genes. Interestingly, in plants, the plant homolog for *HAC1/XBP1* has not yet been reported, but two membrane-associated bZIP transcription factors, bZIP60 and bZIP28, were found to play essential roles in the ER stress response [10], [11].

Recent studies on the UPR pathway of opportunistic pathogenic fungi, including *Aspergillus fumigatus* and *Candida albicans*, reported that the *HAC1* orthologs of these pathogens have an unconventional intron of 20-nt and 19-nt, respectively, which is excised in response to ER stress. Activation of the UPR pathway regulates the expression of genes which function in ER stress, protein secretion, morphological differentiation, and fungal virulence [12], [13]. In contrast to the ascomycetous fungal pathogens, here, we report the unique features of the UPR pathway in controlling growth, ER-stress response, and virulence of a basidiomycetous human fungal *Cryptococcus neoformans*, which causes fatal meningoencephalitis in both immunocompromised and immunocompetent individuals. A recent epidemiological study reported that cryptococcosis is responsible for more than 600,000 deaths annually in AIDS patients [14]. The present study demonstrates that the UPR pathway of *Cryptococcus* is composed of an evolutionarily conserved Ire1 protein kinase and a novel bZIP transcription factor, Hxl1, which is very divergent from yeast Hac1 and human Xbp1. In response to various stresses, the *HXL1* mRNA is subjected to the Ire1-mediated unconventional splicing, which is essential for thermotolerance, cell wall integrity, antifungal drug susceptibility, and virulence of *C*. *neoformans*. Hence this study proposes components of the UPR pathway, particularly Hxl1, as one of ideal targets for development of novel antifungal therapies.

## Results

### *IRE1* plays a critical role in ER-stress response, cell wall integrity, and thermotolerance in *C. neoformans*

**Figure 1:**
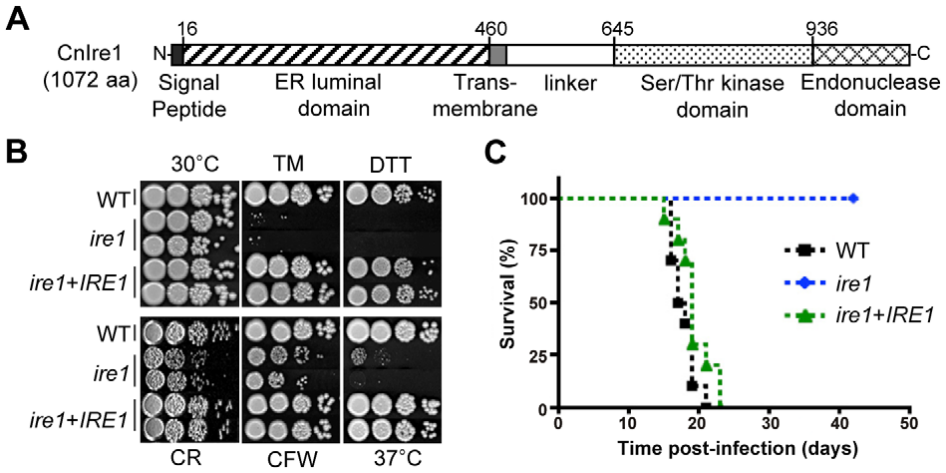
Functional analyses of *C. neoformans ire1* strains. (A) The conserved domain structure of the *C. neoformans* Ire1 protein. (B) For sensitive test to UPR- and cell wall- stresses, wild-type (H99), *ire1* (YSB552 and YSB554), and *ire1*+*IRE1* (YSB1000 and YSB1005) strains were serially diluted and spotted on YPD media with or without exposure to ER stress agents and cell wall disturbing agents (0.075 µg/ml TM, 10 mM DTT, 5 mg/ml CR, 1.5 mg/ml CFW) and incubated at 30°C for 3.5 days. (C) Virulence analysis of *ire1* strains. A/Jcr mice were infected with 10^5^ cells of *MAT*α WT (black square, H99), *ire1* (blue diamond, YSB552), *ire1+IRE1* complemented (green triangle, YSB1000) strains by intranasal inhalation. Percent survival (%) was monitored for 6 weeks post-infection. *P*<0.0001 for WT vs. *ire1* mutant and *P* = 0.1001 for WT vs. *ire1*+*IRE1* complemented strain.

To elucidate the role of the UPR pathway in *C. neoformans*, we first identified and characterized the function of the *C. neoformans* Ire1 ortholog. By BLASTp search using the protein sequence of *S. cerevisiae* Ire1 (ScIre1) as a query, we identified a single gene (CNAG_03670.2), which is highly homologous to ScIre1 (25% identity in amino acid sequence) in the genome database of the serotype A *C. neoformans* strain H99. The CNAG_03670.2 gene encodes a protein of 1,072 amino acids and contains typical Ire1-domain structures such as a luminal domain, a Ser/Thr protein kinase, and a ribonuclease domain (Figure 1A). Based on the significant sequence homology, we named it *IRE1* in *C. neoformans*.

To functionally characterize the role of Ire1 in *C. neoformans*, we constructed *ire1* mutants and *ire1*+*IRE1* complemented strains in the *C. neoformans* H99 strain background (Table 1 and S1 and Figure S1). First we examined whether Ire1 plays a role in ER-stress response by testing sensitivity to tunicamycin (TM) and dithiothreitol (DTT), the well-known ER-stress inducing agents [5]. The *ire1* mutants exhibited considerably increased sensitivity to both TM and DTT compared to the wild-type and their complemented strains (Figure 1B). Moreover, as reported in UPR-defective mutants of other yeast and fungi [12], [13], [15], [16], the *ire1* mutants also exhibited hypersensitivity to both cell wall damaging agents, such as Congo red (CR) and Calcofluor white (CFW) (Figure 1B).

**Table 1:** Strains used in this study.

Strain Name	Genotype	Parental strain	Reference
*C. neoformans*			
H99	Serotype A *MAT*α		[48]
KN99	Serotype A *MAT* **a**		[49]
JEC20	Serotype D *MAT*α		[50]
JEC21	Serotype D *MAT* **a**		[50]
YSB552	*MAT*α *ire1::NAT-STM*#224*	H99	This study
YSB554	*MAT*α *ire1::NAT-STM*#224	H99	This study
YSB723	*MAT*α *hxl1::NAT-STM*#229	H99	This study
YSB730	*MAT*α *hxl1::NAT-STM*#229	H99	This study
YSB1000	*MAT*α *ire1::NAT IRE1-NEO*	YSB552	This study
YSB1005	*MAT*α *ire1::NAT IRE1-NEO*	YSB554	This study
YSB1125	*MAT*α *ire1::NAT HXL1-HXL1* ^u^ *-NEO*	YSB552	This study
YSB743	*MAT*α *ire1::NAT HXL1-HXL1* ^u^ *-NEO*	YSB554	This study
YSB1127	*MAT*α *ire1::NAT HXL1-HXL1* ^s^ *-NEO*	YSB552	This study
YSB747	*MAT*α *ire1::NAT HXL1-HXL1* ^s^ *-NEO*	YSB554	This study
YSB762	*MAT*α *hxl1::NAT HXL1* ^u^ *-NEO*	YSB723	This study
KK1	*MAT*α *cna1::NAT-STM#117*	H99	[51]
KK3	*MAT*α *mpk1:: NAT-STM#150*	H99	[51]
YSB42	*MAT*α *cac1::NAT-STM#159*	H99	[41]
YSB53	*MAT*α *ras1:: NAT-STM#150*	H99	[41]
YSB64	*MAT*α *hog1::NAT-STM#177*	H99	[45]
YSB127	*MAT*α *cpk1::NAT-STM#184*	H99	[52]
YSB736	*MAT*α*-HXL1* ^u^ *-NEO*	H99	This study
YSB741	*MAT*α*- HXL1* ^s^ *-NEO*	H99	This study
YSB1320	*MAT*α *cna1::NAT-STM#117 HXL1* ^u^ *-NEO*	KK1	This study
YSB1325	*MAT*α *cna1::NAT-STM#117 HXL1* ^s^ *-NEO*	KK1	This study
*C. gattii*			
R265	Serotype B *MAT*α		[53]
WM276	Serotype B *MAT*α		[54]
*S. cerevisiae*			
BY4742	*MAT*α *his3 leu2 lys2 ura3*		Open Biosystems
15650	*MAT*α *his3 leu2 lys2 ura3 hac1::KanMX*		Open Biosystems
SCH1	*MAT*α *his3 leu2 lys2 ura3 HAC1::CnHXL1* ^u^		This study
SCH2	*MAT*α *his3 leu2 lys2 ura3 HAC1::CnHXL1* ^s^		This study
SCH3	*MAT*α *his3 leu2 lys2 ura3 hac1::KanMX hac1::CnHXL1* ^u^		This study
SCH4	*MAT*α *his3 leu2 lys2 ura3 hac1::KanMX hac1::CnHXL1* ^s^		This study

*Each *NAT-STM#* indicates the Nat^r^ marker with a unique signature tag.

Another notable phenotype of the *ire1* mutant was its extreme thermosensitivity. The *ire1* mutant was unable to grow at 37°C, which is host physiological temperature (Figure 1B). This finding prompted us to investigate the virulence of the *ire1* mutant. Notably, the *ire1* mutant was avirulent in a murine model of systemic cryptococcosis (Figure 1C). Even after 42 days post infection, all of the mice infected with the *ire1* mutant remained healthy and asymptomatic. In contrast, the mice infected with the wild-type or *ire1*+*IRE1* complemented strains became ill by 18 to 23 days post infection (Figure 1C). In conclusion, Ire1 plays crucial roles in ER-stress response, cell wall integrity, and thermotolerance of *C. neoformans*.

### *C. neoformans HXL1,* encoding a novel bZIP protein, is subject to Ire1-mediated unconventional splicing

**Figure 2:**
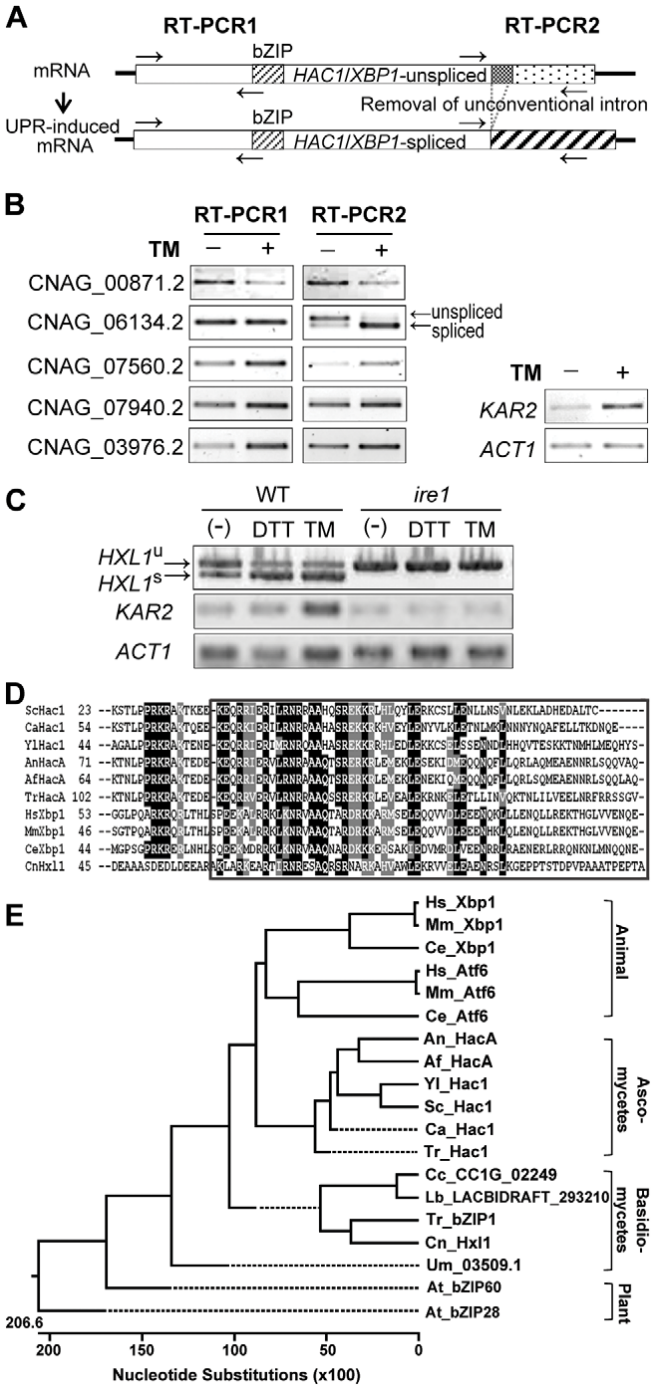
Identification of a novel Ire1-dependent bZIP transcription factor, Hxl1, in *C. neoformans*. (A) General mRNA structures of the unspliced and spliced *HAC1*/*XBP1*. For identification of putative *HAC1*/*XBP1* homologs, primers for RT-PCR1 and RT-PCR2 were designed to amplify the 5′-region product and the unconventionally spliced 3′-region product, respectively. (B) RT-PCR analysis for testing UPR-induced splicing. RT-PCR of CNAG_00871.2, CNAG_06134.2, CNAG_07560.2, CNAG_07940.2, CNAG_03976.2, *KAR2* (CNAG_06443.2), and *ACT1* (CNAG_00483.2) were performed with the cDNA samples of H99 strain cultivated in YPD medium with or without TM (5 µg/ml) treatment for 1 hr. (C) *IRE1*-dependent splicing of CNAG_06134.2. Strains were cultivated in YPD with or without TM (8 µg/ml) or DTT (20 mM). (D) Alignment of bZIP domains observed in Hxl1 and Hac1/Xbp1 homologs from several fungi and mammals. DNA binding domain of the bZIP domain is boxed. (E) Phylogenetic tree of the bZIP domains of transcription factors responsible for ER stress responses or Hxl1 homologs in fungi (ascomycete and basidiomycete), worm, plant, and animals. Hs: *Homo sapiens* Xbp1 (NP_001073007), Atf6 (NP_031374); Mm: *Mus musculus* Xbp1 (AAL60202), Atf6 (NP_001074773); Ce: *Caenorhabditis. elegans* Xbp1 (AAL60201), Atf6 (NP_510094); Sc, *S. cerevisiae* Hac1 (NP_116622); Ca: *C. albicans* Hac1 (EF655649); Yl: *Yarrowia lipolytica* Hac1 (XP_500811); An: *Aspergillus nidulans* hacA, Q8TFU8; Af: *A. fumigatus* HacA, ACJ61678; Tr: *T. reesei* hac1 (Q8TFF3); Cn: *C. neoformans* Hxl1 (CNAG_06134.2); Um: *Ustilago maydis* UM03509.1; Tf: *Tremella fuciformis* bZIP 1 (XP_001877531); Lb: *Laccaria bicolor* LACBIDRAFT_293210; Cc: *Coprinopsis cinerea* CC1G_02249; At: *Arabidopsis thaliana* bZIP28 (NP_187691), bZIP60 (NP_174998).

In the conventional UPR signaling pathway, Ire1 activates its downstream transcription factor Hac1/Xbp1 upon ER-stress by removing an unconventional intron at the 3′-region of *HAC1*/*XBP1* mRNA (Figure 2A). Therefore, we searched for the yeast *HAC1* or human *XBP1* ortholog. Interestingly, however, at the initial BLASTp search, no *C. neoformans* gene showed significant sequence homology to either full-length *HAC1*/*XBP1*, or to just the isolated bZIP domain that is relatively highly conserved among Hac1/Xbp1 orthologs. By lowering the stringency in BLASTp search (e-value  = 10^−2^) and using only the bZIP domain sequences of Hac1/Xbp1, five putative genes were selected with a limited sequence homology to the Hac1/Xbp1 proteins (Table S2).

To identify a *HAC1*/*XBP1*-like gene in *C. neoformans* that would undergo a typical ER-stress induced unconventional splicing, we screened for splicing events in the 3′-regions of each mRNA by RT-PCR after TM treatment, which was shown to induce the expression of *C. neoformans KAR2* (ER protein chaperone BiP) (Figure 2B), a well-known ER stress defense gene in yeasts [4]. Among the candidate genes, only CNAG_06134.2 exhibited two RT-PCR products with different sizes in the 3′-region (RT-PCR2), but a single RT-PCR product in the 5′-region (RT-PCR1) under unstressed conditions. In response to ER stress, the smaller RT-PCR2 products became more prominent (Figure 2B). Other candidate bZIP proteins did not appear to undergo unconventional splicing events although their expression levels were differentially regulated in response to ER stress. Next we examined the occurrence of unconventional splicing of CNAG_06134.2 in the *C. neoformans ire1* mutant strain. CNAG_06134.2 did not show unconventional splicing event in the *ire1* mutant strain in response to both TM and DTT (Figure 2C), indicating that the CNAG_06134.2 mRNA is a downstream target of Ire1 in the UPR signaling pathway of *C. neoformans*. Sequence analysis of the RT-PCR2 product of CNAG_06134.2 revealed that the nonconventional 56-nt intron was removed upon UPR induction. Therefore, we named CNAG_06134.2 *HXL1* (*HAC1* and *XBP1*-*L*ike gene *1*). Interestingly, about 20-30% of *C. neoformans HXL1* mRNA was detected as a spliced form (*HXL1* ^u^) even under normal, unstressed condition, whereas more than 80% of *HXL1* mRNA was converted to a spliced form (*HXL1* ^s^) after DTT or TM treatment (Figure 2C and S3), indicating that the UPR pathway appears to be partly activated by normal *in vitro* culture conditions without ER stress in *C. neoformans*. The increased relative ratio of *HXL1* ^s^ to *HXL1* ^u^ form of *HXL1* mRNA upon ER stress showed a good correlation with the increased level of *KAR2* transcript, suggesting that the *HXL1* mRNA splicing event activated the UPR pathway. As shown in multiple sequence alignment and phylogenetic tree analysis of bZIP domains, the protein product of *HXL1* (Hxl1) is only distantly related to known Hac1/Xbp1 transcription factors (Figure 2D and E), indicating that *C. neoformans* contains both evolutionarily conserved and distinct signaling components, Ire1 and Hxl1, respectively, for the UPR pathway.

**Figure 3:**
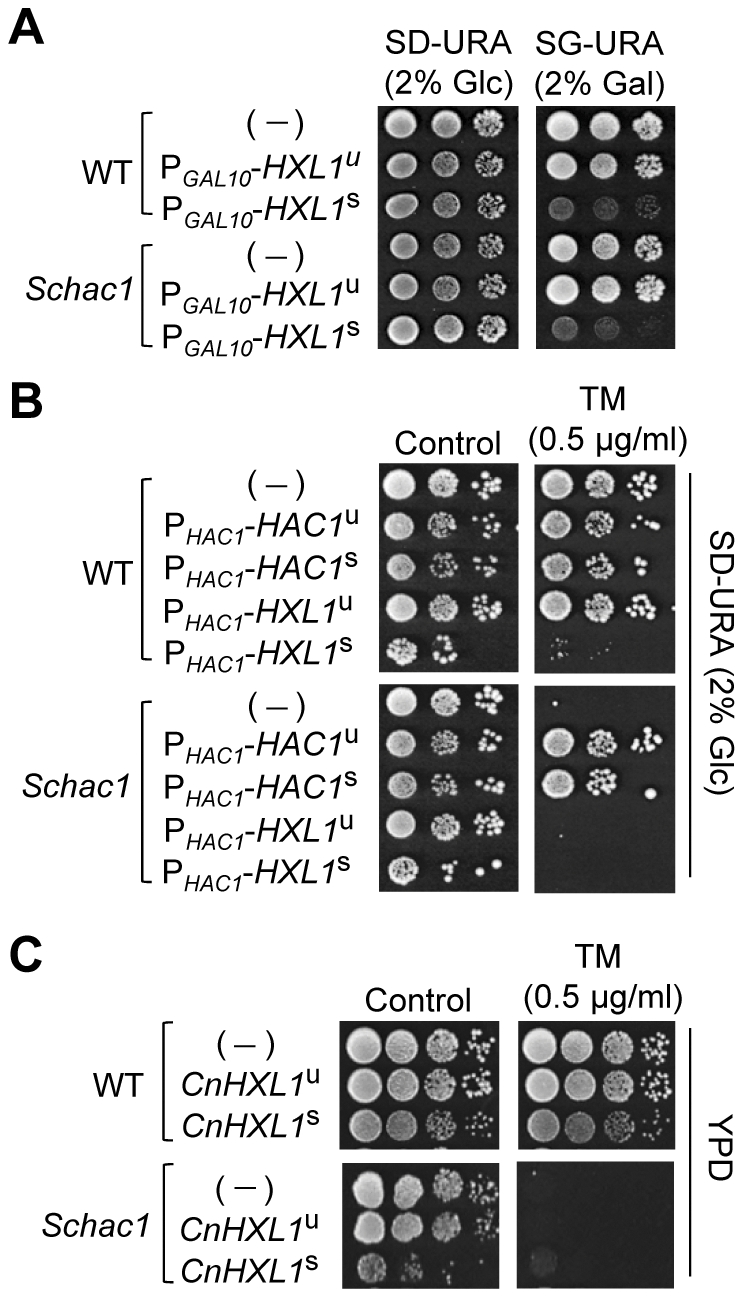
Heterologous expression analysis of *C. neoformans HXL1* in *S. cerevisiae hac1* mutants. (A) Overexpression of *C. neoformans HXL1* by the *GAL10* promoter. *S. cerevisiae* wild type and *hac1* strains harboring the expression vectors of unspliced or spliced forms of *CnHXL* under the *GAL10* promoter (P*_GAL10_-HXL1^u^* and P*_GAL10_-HXL1* ^s^, respectively) were serially diluted and spotted on synthetic minimal media containing 2% glucose (repression) or 2% galactose (induction). (B) Expression of *CnHXL1* by the *ScHAC1* promoter. Wild- type and *Schac1* strains harboring the expression vectors of unspliced or spliced forms of *ScHAC1* (P*_HAC1_-HAC1* ^u^ and P*_HAC1_-HAC1* ^s^, respectively) and unspliced or spliced forms of *CnHXL1* under the *ScHAC1* promoter (P*_HAC1_-HXL1* ^u^ and P*_HAC1_-HXL1* ^s^, respectively) were serially diluted and spotted on synthetic minimal media with or without TM. Strains were incubated at 30°C for 3 days and photographed. (C) Integrative expression of *CnHXL1.* Wild-type (BY4742) and *Schac1* strains harboring a single copy of unspliced or spliced forms of *CnHXL1*integrated into the *ScHAC1* locus were serially diluted and spotted on complex YPD media with or without TM. Strains were incubated at 30°C for 2 days and photographed.

Since the Hxl1 is structurally and phylogenetically distant from the Hac1/Xbp1, we addressed whether Hxl1 can complement the TM sensitivity of *S. cerevisiae hac1* (*Schac1*) strain. *S. cerevisiae* wild type (BY4742) and *Schac1* mutant strains were transformed with low copy expression vectors containing *C. neoformans* unspliced form of *HXL1* or spliced form of *HXL1*. Overexpression of the spliced *HXL1* ^s^ controlled by the *GAL10* promoter led to severe growth inhibition even in wild type as well as in *Schac1* strains (Figure 3A). Contrast to the *ScHAC1* ^u^ or *ScHAC1* ^s^ expression, the *HXL1* ^s^ expression driven by the *ScHAC1* promoter could not recover the TM sensitivity (Figure 3B) and the inositol auxotrophy of *Schac1* strain (data not shown), and caused rather growth inhibition even in wild type under normal conditions (Figure 3B). In agreement with these data, a single copy integration of the *CnHXL1* ^s^ gene into the *ScHAC1* locus did not recover the TM sensitivity of *Schac1* mutant (Figure 3C). This is in contrast to the effective compensation for the loss of *HAC1* in *S. cerevisiae* by ascomycetes *Trichoderma reesei* or *C. albicans* Hac1 homologs [12], [17]. These results strongly suggest that the *C. neoformans* Hxl1 may not function properly with the *S. cerevisiae* transcriptional machinery due to its unique structures.

### Structural conservation of *HXL1* in different serotypes of *C. neoformans*

**Figure 4:**
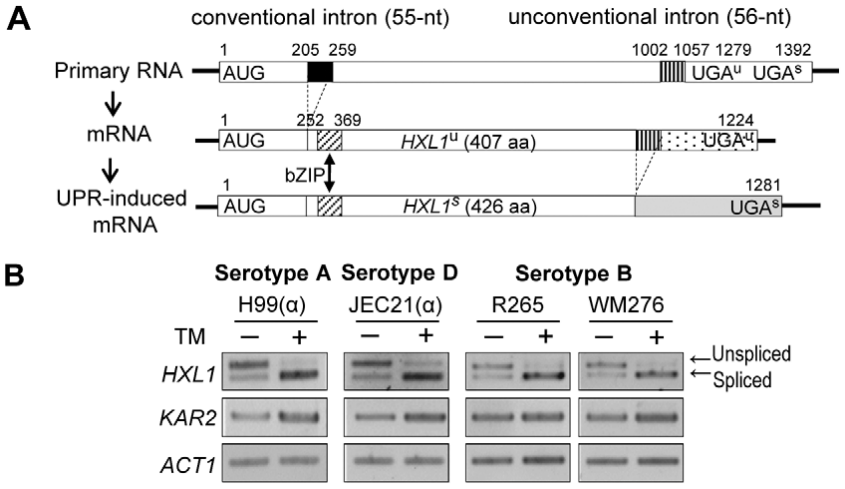
Structural conservation of the distinct *HXL1* gene in different serotypes of *C. neoformans.* (A) Scheme for conventional and UPR-induced non-conventional splicing of *C. neoformans HXL1.* The correct frame of the bZIP domain is formed after conventional splicing of the *HXLI* primary mRNA and shown as a diagonal box. The conventional and unconventional introns are shown as a solid black box and a vertical striped box, respectively. The C-terminal fragments of *HXL1* before and after unconventional splicing are shown as the dotted box and a grayed box, respectively. (B) RT-PCR analysis of UPR-induced splicing of *HXL1* in *C. neoformans* strains of different serotypes. The cDNA samples, synthesized from yeast cells treated with or without TM (5 µg/ml) for 1 hr, were used as templates for RT-PCR of *HXL1.*

Because Hxl1 was identified in the serotype A *C. neoformans* strain H99 and found to be phylogenetically distinct from other Hac1/Xbp1 orthologs, we addressed whether *HXL1* is present in other serotypes of *C. neoformans.* Serotype D strains (JEC21 and B-3501A) contain an *HXL1* ortholog (CNM01380 and CNBM1240, respectively) and the serotype B strains (R265 and WM276) also contain an *HXL1* ortholog (CNBG_4842.2 and CGB_M1590W, respectively). Sequence analysis of *HXL1* revealed that all of the *HXL1* genes of different serotypes have both a conventional intron of 55-nt and the unconventional intron of 56-nt at the upstream and downstream regions of the bZIP domain, respectively, as observed in the serotype A strain H99 (Figure 4A). All of the *C. neoformans* strains also exhibited UPR-induced unconventional splicing of *HXL1* mRNA (Figure 4B), indicating that the structural organization of *HXL1* is widely conserved in different serotypes of *C. neoformans*.

Although the sequences of *HXL1* were not completely identical, the position and size of unconventional intron and its border sequences were well conserved in all serotype strains of *C. neoformans* (Figure S2A). It is noteworthy that the *HXL1* ortholog of serotype B (*Cryptococcus gattii*) encodes a protein of 406 (unspliced form) and 426 (spliced form) amino acids, respectively, due to loss of an alanine residue caused by a 3-bp deletion (CGC, +144 to +146) compared with those of serotype A and D strains.

### Hxl1 functions as an essential downstream transcription factor of Ire1

**Figure 5:**
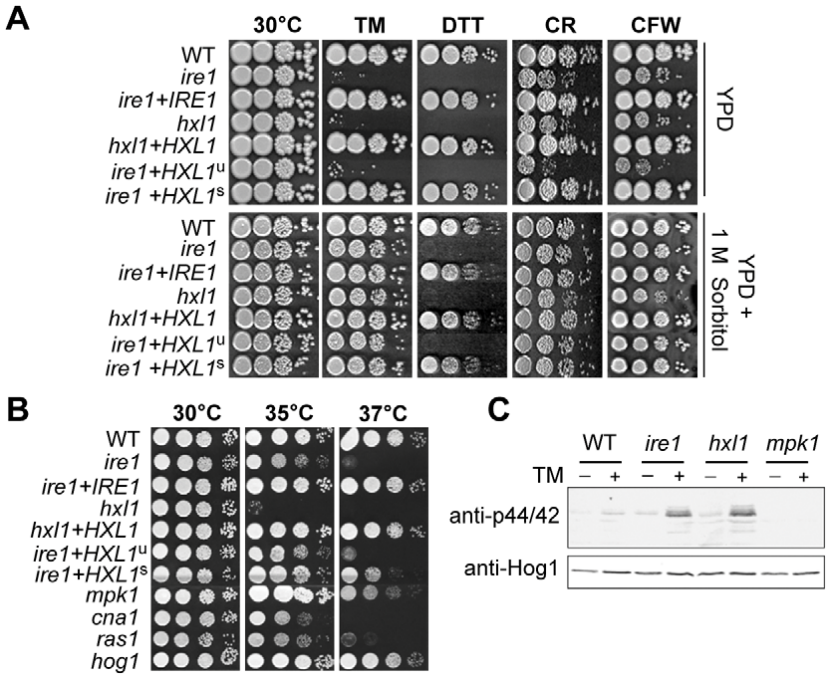
Functional analyses of *C. neoformans HXL1* deletion and complementation. (A) For sensitive test to UPR- and cell wall- stresses, strains were serially diluted and spotted on YPD media with or without exposure to ER stress agents and cell wall disturbing agents (0.075 µg/ml TM, 10 mM DTT, 5 mg/ml CR, 1.5 mg/ml CFW), without (top panel) or with 1 M sorbitol (bottom panel) and incubated at 30°C for 3.5 days. (B) For the thermosensitivity test, strains were spotted on YPD medium and incubated at 35°C or 37°C for 3.5 days. (C) Phosphorylation of Mpk1 induced by ER stress and loss of UPR function. Strains in early exponential phase were treated with TM (5 µg/ml) at 30°C for 2 hr. 30 µg of total protein was analyzed by Western blotting with phospho-p44/42-MAPK antibody (upper panel) for the phosphorylated Mpk1 (Mpk1-P) protein and anti-Hog1 antibody (lower panel) for the Hog1 protein as a loading control. Strains were: wild-type (H99), *ire1* (YSB552), *ire1+IRE1* complemented (YSB1000), *hxl1* (YSB723), *hxl1*+*HXL1* ^u^ complemented (YSB762), *ire1*+*HXL1* ^u^ suppressed (YSB743), *ire1*+*HXL1* ^s^ suppressed (YSB747), *mpk*1 (KK3), *cna1* (KK1), *ras1* (YSB53), and *hog1* (YSB64).

To analyze whether Hxl1 is the target transcription factor of Ire1, we constructed *hxl1* mutant strains (Table 1 and Figure S1) and compared their phenotypes with those of the *ire1* mutants. Similar to the *ire1* mutant, *hxl1* mutants exhibited equivalent levels of sensitivity to TM, DTT, CR, or CFW (Figure 5A). Verifying these results, targeted re-integration of the *HXL1* into its native locus (the *hxl1*+*HXL1* strain) completely restored wild-type levels of resistance of the *hxl1* mutant to ER stress-inducing and cell wall disturbing agents. Most importantly, the UPR-induced spliced form of *HXL1* (*HXL1* ^s^), but not the unspliced form of *HXL1* (*HXL1* ^u^), completely suppressed ER stress sensitivity of the *ire1* mutant to the same extent observed in the wild-type and *ire1*+*IRE1* complemented strains (Figure 5A), demonstrating that Hxl1 functions downstream of Ire1. Remarkably, the *hxl1* mutant displayed a more severe growth defect than the *ire1*, *cna1*, *ras1*, and *mpk1* strains at high temperature (Figure 5B).

TM sensitivity of the UPR mutants was fully recovered by addition of an osmotic stabilizer 1 M sorbitol, whereas DTT sensitivity was not, indicating that TM and DTT evoke ER stress in different ways (Figure 5A). TM treatment would mainly result in the defect in *N*-glycosylation, affecting mainly cell wall integrity, whereas DTT could act as oxidative damaging agent. Supporting this, the hypersensitivity to cell wall destabilizers of both UPR mutant strains of *C. neoformans* was mostly rescued by 1 M sorbitol (Figure 5A). Notably the basal level phosphorylation of the cell wall integrity-controlling Mpk1 MAPK was about threefold higher in the *ire1* and *hxl1* mutants compared to the wild-type even during normal growth. The levels of phosphorylated Mpk1 increased much more in the UPR mutant strains compared to the wild-type upon TM treatment (Figure 5C). Thus defects in the UPR pathway and/or exposure to ER stress generate cell wall defects, which trigger induction of the Mpk1-mediated cell wall integrity pathway in *C. neoformans*, as previously shown in *S. cerevisiae* [15], [18]. Taken together, inactivation of the Ire1-Hxl1-mediated UPR pathway may cause extreme thermosensitivity of *C. neoformans* not only via a cell wall integrity defect but also due to defects in several other biological processes.

### Subsets of UPR genes are differentially regulated by Ire1 and Hxl1

**Figure 6:**
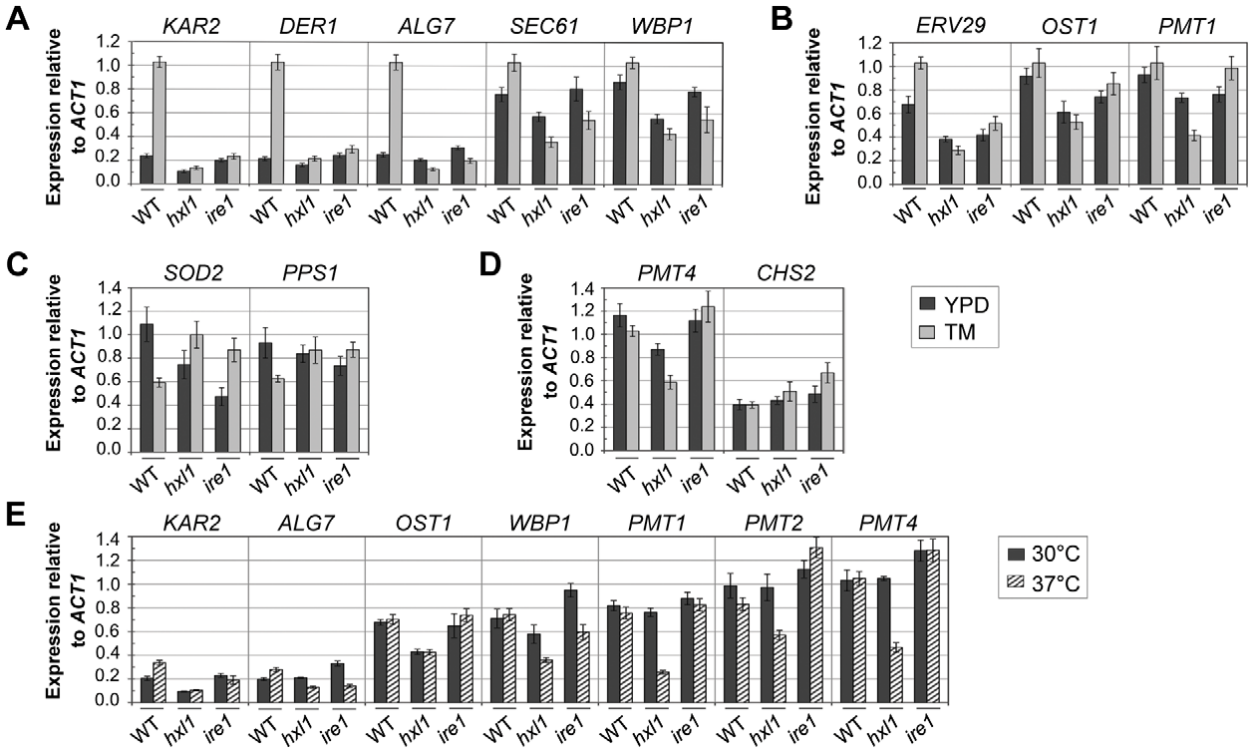
Quantitative real-time PCR analysis of Hxl1-dependent regulation of putative UPR genes. Strains were grown in YPD medium to early exponential phase, exposed to TM (5 µg/ml) for 1 hr (A–D) or to high temperature at 37°C for 2 hr (E), and harvested. The relative expression levels of putative UPR-regulated genes were analyzed by qRT-PCR with primers listed in Table S1 and normalized to that of *ACT1*. Strains were wild-type (H99), *hxl1* mutant strain (YSB723), and *ire1* mutant (YSB552). Error bars represent standard deviation (SD) of triplicated (A–D) or duplicated (E).

To verify further Hxl1 as a transcription factor of the UPR pathway in *C. neoformans*, we measured the transcript levels of putative UPR-regulated genes under ER stress conditions. A set of *C. neoformans* homologs of the *S. cerevisiae HAC1* regulons (Figure S4) harboring the UPRE and UPRE-like sequences (CAGNGTG, TGACGTCA, CCAGC, CGTGTCGG, or TACGTG) [19], [20] in their promoters, were chosen and their transcript levels were compared between the wild-type and the UPR mutant strains after TM treatment. The *C. neoformans* homologs of well-established UPR target genes involved in protein secretion and glycosylation, such as *KAR2*, *SEC61* (translocation of misfolded proteins out of ER), *DER1* (ER-associated degradation), *WBP1* (lipid-linked *N*-oligosaccharyltransferase), and *ALG7* (lipid-linked *N*-oligosaccharide biosynthesis), were up-regulated upon TM treatment in an Ire1- and Hxl1-dependent manner (Figure 6A). However, it is notable that the induced expression of some UPR target candidates such as *ERV29* (ER to Golgi vesicle-mediated transport), *OST1* (lipid-linked *N*-oligosaccharyltransferase), and *PMT1* (protein *O*-mannosyltransferase) homologs were dependent on Hxl1 but not on Ire1 (Figure 6B). On the other hand, the *SOD2* (mitochondrial superoxide dismutase) and *PPS1* (protein phosphatase S phase) homologs, which are responsive to osmotic and heat stress in *C. neoformans*, respectively [21], [22], were down-regulated under ER stress in an Ire1- and Hxl1-dependent manner (Figure 6C). For some *C. neoformans* genes such as *PMT4* (protein *O*-mannosyltransferase) and *CHS2* (chitin biosynthesis), their expression levels did not change in the wild-type or *hxl1* mutants during ER stress, but significantly increased in the *ire1* mutant (Figure 6D). All these results strongly indicate that although Hxl1 and Ire1 mainly work together in the UPR pathway, the loss of their function can generate differential effect on the expression of some subsets of UPR genes in response to ER stress.

It is noteworthy that the *hxl1* mutant showed a much more severe thermosensitive phenotype than the *ire1* mutant and that the introduction of *HXL1* ^s^ did not completely suppress the thermosensitivity conferred by *ire1* mutation (Figure 5B). To understand the differences with respect to thermotolerance, we also analyzed the expression patterns of the putative UPR genes under heat stress (Figure 6E). *KAR2* and *ALG7* showed an induced expression pattern upon heat stress in an Ire1- and Hxl1-dependent manner. Intriguingly, however, genes involved in protein *N*-/*O*-glycosylation, such as *WBP1*, *PMT1*, *PMT2*, and *PMT4*, showed about twofold decreased expression only in the *hxl1* mutant, but not in either the wild-type or the *ire1* mutant (Figure 6E). These results indicate that compared to the loss of Ire1, the absence of Hxl1 might exert more negative effect on expression of some UPR genes under heat stress, partly explaining why the *hxl1* mutant was more thermosensitive than the *ire1* mutant.

### Ire1 and Hxl1 play similar and distinct roles in controlling virulence and antifungal drug resistance of *C. neoformans*

**Figure 7:**
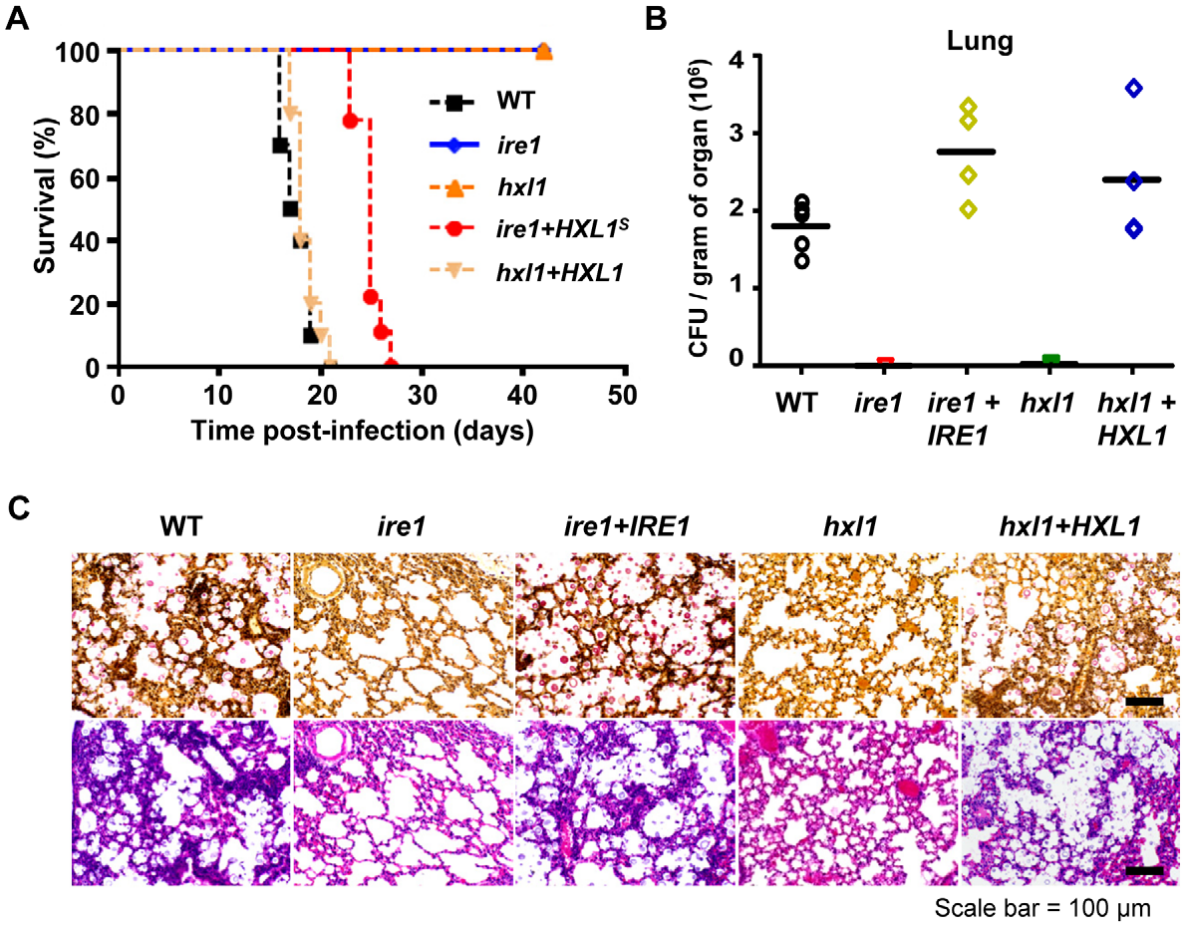
The *IRE1*-*HXL1* UPR pathway is essential for virulence of *C. neoformans*. (A) A/Jcr mice were infected with 10^5^ cells of *MAT*α WT (black square, H99), *ire1* (blue diamond, YSB554), *hxl1* (orange triangle, YSB723), *ire1*+*HXL1* ^s^ suppressed (red circle, YSB747), and *hxl1*+*HXL1* ^u^ complemented strains (peach inverted triangle, YSB762) by intranasal instillation. Survival (in %) was monitored for 6 weeks post-infection (*P*<0.0001 for WT vs. *ire1*, WT vs. *hxl1*, WT vs. *hxl1*+*HXL1* ^u^, and WT vs. *ire1*+*HXL1* ^s^ strains). (B) The colony forming units (CFU) of surviving fungal cells per gram of organ was determined in the lung from sacrificed animals infected with WT (H99), *ire1* (YSB552), *ire1*+*IRE1* (YSB1000), *hxl1* (YSB723), and *hxl1*+*HXL1* (YSB762) (*P*<0.05 for WT vs. *ire1* and WT vs. *hxl1*, *P*>0.05 WT vs. *ire1*+*IRE1* and WT vs. *hxl1*+*HXL1* strains). (C) Histopathological staining of infected lung tissues with mucicarmine (upper panel) and H&E (lower panel).

We further examined whether Hxl1p also plays a critical role in virulence of *C. neoformans* like Ire1p. As similar to the *ire1* mutant, the *hxl1* mutant was avirulent in a murine model of systemic cryptococcosis and their virulence was restored by reintroduction of the wild-type genes (Figure 7A). To ensure that Ire1 controls virulence of *C. neoformans* via its downstream Hxl1, we also analyzed mice infected with an *ire1* strain harboring the activated, spliced *HXL1* ^s^. Interestingly, expression of the *HXL1* ^s^ partially restored virulence of the *ire1* mutant strain (Figure 7A). The *ire1*+*HXL1* ^s^ strain was less virulent than the wild-type or the *ire1*+*IRE1* strains, but still retained virulence. Taken together, it is conceivable that Ire1 controls virulence of *C. neoformans* both in Hxl1-dependent and -independent manners.

To elucidate whether the fully attenuated virulence of the *ire1* and *hxl1* mutants is caused by reduced cell survival or proliferation in the host, we performed fungal burden assay to determine the number of *C. neoformans* cells recovered from the sacrificed mice. The *ire1* and *hxl1* mutants showed significant decrease in the cell numbers compared to wild-type in the lung and brain tissues (Figure 7B and S5). Furthermore, the histopathological analysis of mucicarmine-stained tissues revealed that the wild-type (H99), *ire1*+*IRE1*, and *hxl*+*HXL1* complemented strains proliferate extensively in lung tissues, while cells of the *ire1* and *hxl1* mutants were not observed (Figure 7C), suggesting that Ire1 and Hxl1 are required for proliferation in lung tissues. In the H&E staining, alveolar damage or tissue necrosis in lung tissues was observed only in mice infected with the wild type and *ire1*+*IRE1* and *hxl*+*HXL1* complemented strains but not with the *ire1* or *hxl1* mutants (Figure 7C). The result of histopathological analysis was well correlated with that of fungal burden in the lung tissues. However, in the brain tissues, we did not observe mucicarmine-stained or damaged tissues in animals infected with any strains (data not shown). Taken together, the results showed that the UPR pathway is essential for *C. neoformans* to survive and proliferate during infection.

**Figure 8:**
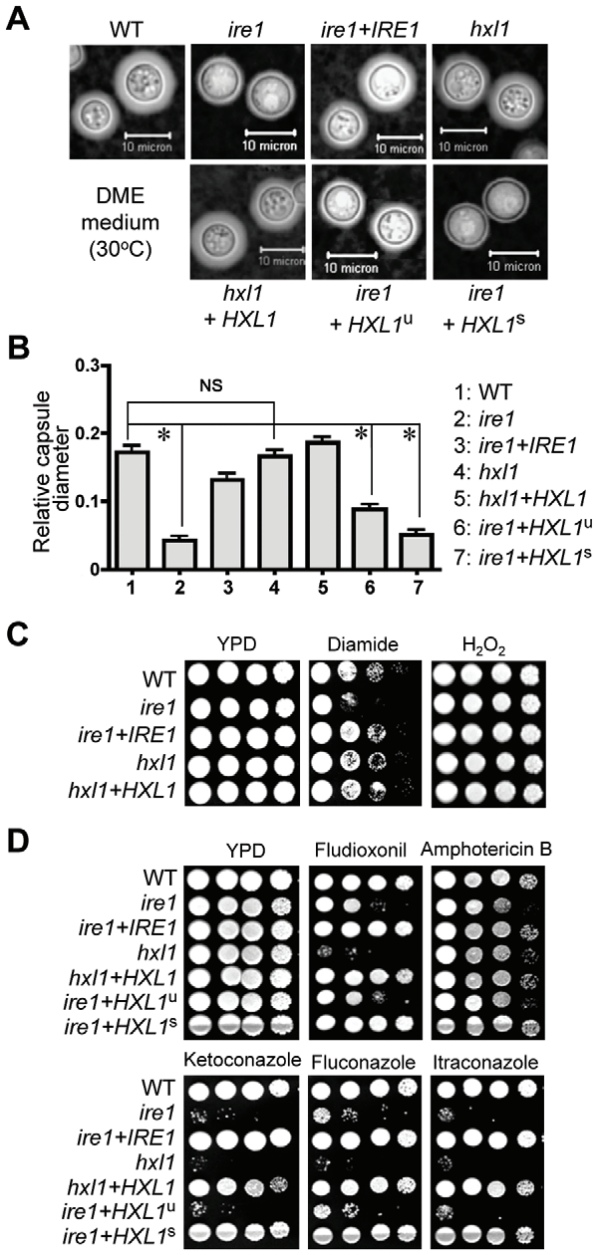
Ire1 controls capsule formation, oxidative stress responses, and antifungal drugs resistance in Hxl1 dependent- and independent manners in *C. neformasn*. (A and B) Strains lacking *IRE1* produce less capsule. Strains were grown at 30°C on solid DME medium for 2 days and photographed. The relative capsule diameter of cells was measured after India ink staining [*P<0.001 for WT vs. *ire1* mutant, for WT vs. *ire1*+*HXL1* ^u^ mutant, and WT vs. *ire1*+*HXL1^s^* mutant and NS, not significant (*P*>0.05)]. (C) Different sensitivity of *ire1* and *hxl1* mutant strains to diamide. Strains were serially diluted and spotted on YPD medium with 2 mM diamide or 3 mM hydrogen peroxide and incubated at 30°C for 3 days. (D) The UPR is involved in antifungal drug susceptibility. Strains were serially diluted and spotted on YPD medium containing the indicated concentrations of antifungal drugs including ketoconazole (0.1 µg/ml), fluconazole (14 µg/ml), itraconazole (0.5 µg/ml), fludioxonil (0.5 µg/ml), and amphotericin B (0.8 µg/ml). Cells were incubated in 30°C for 3 days. Strains were: WT (H99), *ire1* (YSB552), *ire1*+*IRE1* complemented (YSB1000), *hxl1* (YSB723), *hxl1*+*HXL1* ^u^ complemented (YSB762), *ire1*+*HXL1* ^u^ suppressed (YSB1125), and *ire1*+*HXL1* ^s^ suppressed (YSB1127).

Next we investigated other virulence factors Ire1 might control in an Hxl1p-independent manner. Besides thermotolerance, antioxidant melanin and antiphagocytic capsule are two major virulence factors for *C. neoformans*. Intriguingly, only the *ire1* mutants were defective in capsule biosynthesis whereas *hxl1* mutants were as efficient in capsule production as the wild-type strain based on both qualitative and quantitative measurements (Figure 8A and B). Moreover, the *ire1* mutants with a spliced *HXL1* allele (*ire1+HXL1* ^s^) or an unspliced allele (*ire1+HXL1* ^u^) also exhibited a defect in capsule production. In contrast to capsule biosynthesis, the *ire1* and *hxl1* mutants both produced wild-type levels of melanin (data not shown). We also tested oxidative stress responses of the UPR mutants because the ability to counteract reactive oxygen species is one of key virulence factors in *C. neoformans* [23], [24]. The UPR mutants were as resistant to hydrogen peroxide (H_2_O_2_) as the wild-type strain (Figure 8C). Interestingly, however, the *ire1* mutant, but not the *hxl1* mutant, exhibited increased sensitivity to another oxidant diamide (Figure 8C). The results suggest that Ire1 controls capsule synthesis and diamide-resistance in an Hx1l-independent manner.

Finally we examined the potency of Ire1 or Hxl1 as a target for combination therapy with currently available clinical drugs for treatment of cryptococcosis. There are also reports that *A. fumigatus hacA* or *C. albicans ire1* mutations enhance activity of antifungal drugs such as amphotericin B, caspofungin, and azoles [13], [16]. The loss of *IRE1* or *HXL1* caused dramatically enhanced susceptibility to azoles such as ketoconazole, fluconazole and itraconazole, and phenylpyrroles such as fludioxonil, but only slightly increased susceptibility to polyenes such as amphotericin B (Figure 8D). The *ire1* mutants expressing the spliced *HXL1* allele exhibited wild-type levels of antifungal drug sensitivity, indicating that the Ire1-Hxl1-mediated UPR pathway in *C. neoformans* regulates antifungal drug resistance.

### The Ire1-Hxl1-mediated UPR is induced by various environmental cues in *C. neoformans*

**Figure 9:**
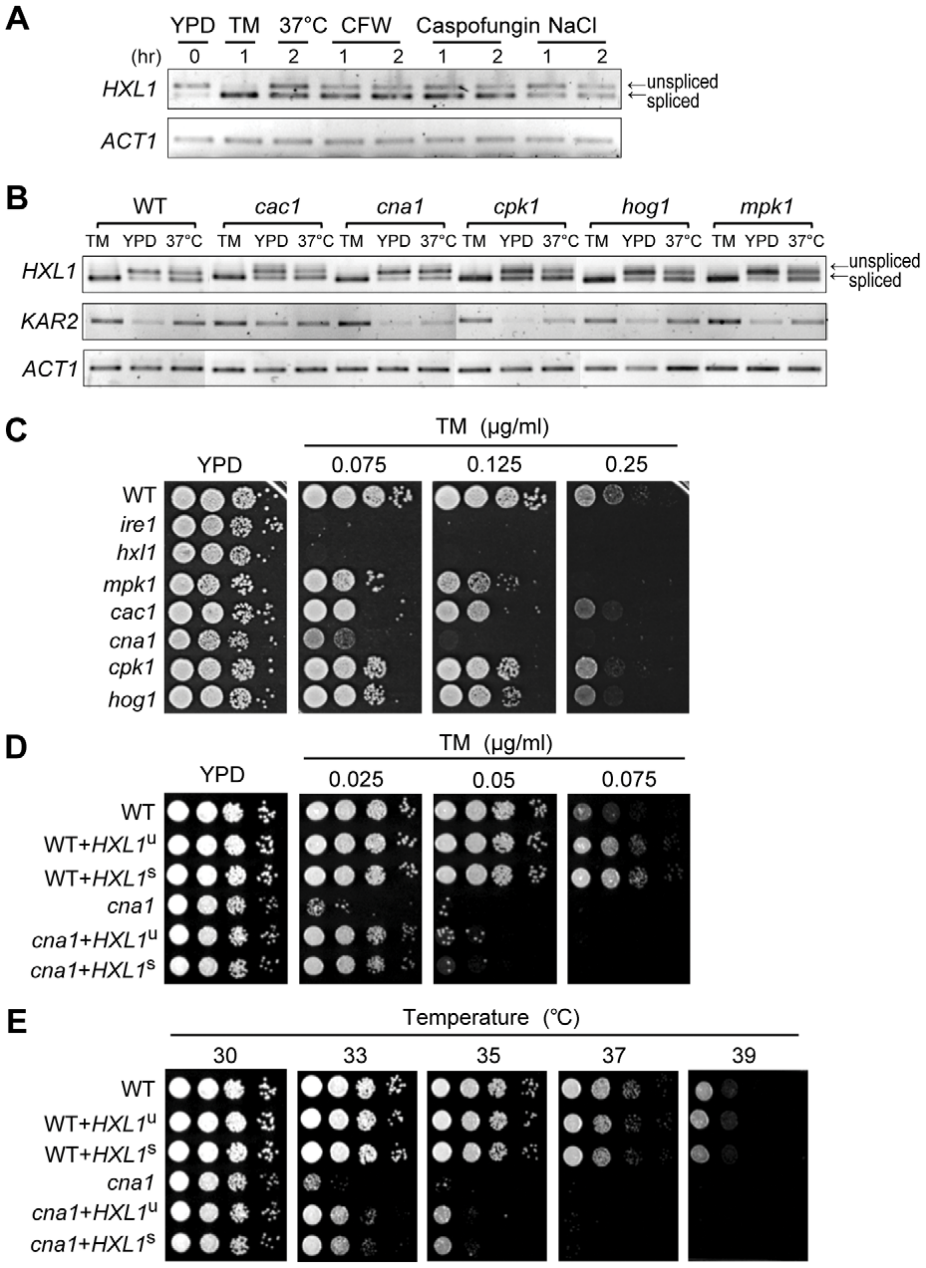
Induction of the UPR pathway by other stresses and potential crosstalk with other signaling pathways. (A) Analysis of the *HXL1* splicing pattern during cell wall and osmotic stresses. RT-PCR of *HXL1* and *ACT1* were performed with the cDNA samples of H99 strain untreated or treated with TM (5 µg/ml), CFW (20 mg/ml), caspofungin (6 µg/ml), and NaCl (1 M) at 30°C, or at 37°C, respectively, for the indicated times. (B) Analysis of the *HXL1* expression pattern in signaling mutants under ER or heat stress conditions. RT-PCR of *HXL1*, *KAR2*, and *ACT1* was performed with cDNA samples of strain H99 untreated or treated with 5 µg/ml TM for 1 hr at 30°C, or at 37°C for 2 hr, respectively. (C) Analysis of ER-stress sensitivity of various signaling mutants. Strains were spotted serially and incubated on YPD media with or without indicated concentrations of TM at 30°C for 2.5 days. (D and E) Phenotypic analyses of WT (H99) and *cna1* mutant strains having increased expression of *HXL1* against TM and thermotolerance. Each strain [WT, WT+*HXL1* ^u^ (YSB736), WT+*HXL1* ^s^ (YSB741), *cna1, cna1*+*HXL1* ^u^ (YSB1320), and *cna1*+*HXL1* ^s^ (YSB1325)] was spotted on YPD medium with indicated concentrations of TM and incubated at 30°C for 3 days for TM sensitivity or was incubated on YPD medium at indicated temperatures for 3 days for thermotolerance test.

To investigate the possible induction of the Ire1-Hxl1-mediated UPR pathway by other environmental stresses and potential crosstalk with other signaling pathways, we examined the activation of *HXL1* splicing under various stress conditions. Besides ER stress, other stress conditions, including treatment with cell wall-disturbing reagents such as CFW or caspofungin, heat shock, and high salt, were shown to activate unconventional splicing of the *HXL1* mRNA, albeit to a lesser extent than TM, in *C. neoformans* (Figure 9A).

This finding led us to examine whether multiple stress-activated signaling pathways play roles in inducing UPR via *HXL1* splicing. In multiple signaling mutant strains we tested, including *cac1* (adenylyl cyclase), *cna1* (calcineurin), *cpk1* (the pheromone responsive MAPK), *hog1* (the stress-activated MAPK), and *mpk1* (the cell wall integrity MAPK), most of *HXL1* ^u^ mRNA was spliced unconventionally in response to TM as in the wild type strain (Figure 9B). This result indicates that cAMP/PKA, calcineurin, and any of MAPK pathways do not directly control the *HXL1* splicing under the ER stress condition in *C. neoformans.* Interestingly, however, under high temperature condition, the ratio of *HXL1* ^s^ to *HXL1* ^u^ apparently decreased in the *cna1* mutants compared to the wild type strain (Figure 9B and S3D). The *cna1* mutant is known to exhibit significant high temperature sensitivity (Figure 5B). Thus these results imply that impairment of the calcineurin signaling pathway can affect regulation of *HXL1* at high temperature directly or indirectly. Furthermore, the induction of *KAR2* was also found to be significantly reduced in the *cna1* mutant (Figure 9B), implicating the potential involvement of the calcineurin pathway in the UPR-mediated thermotolerance. Supporting this, the *cna1* mutant exhibited hypersensitivity to TM, albeit to a lesser extent than the UPR mutant (Figure 9C). Furthermore, increased expression of *HXL1* by integration of *HXL1* ^u^ or *HXL1* ^s^ into the native *HXL1* locus clearly increased TM resistance in both wild-type and *cna1* mutant strains, suggesting that the UPR pathway work parallel with the calcineurin pathway for ER stress response and adaptation (Figure 9D). In contrast, the increased expression of *HXL1* ^u^ or *HXL1* ^s^ partially recovered thermotolerance of the *cna1* mutants, without any positive effect on wild-type strains (Figure 9E), suggesting that the defective thermotolerance caused by inhibition of the calcineurin pathway may partly result from insufficient induction of the UPR pathway. All these data strongly support the potential interaction between the calcineurin and UPR pathways in *C. neoformans*. Taken together, our data suggest that besides ER stress, other stress conditions could also activate the *IRE1*-*HXL1*-mediated UPR via regulation of the unconventional splicing of *HXL1* mRNA in *C. neoformans.*

## Discussion

In eukaryotic cells, protein folding and secretion in the ER is tightly regulated by the ER quality control system, including ER-associated degradation (ERAD) and *N*-glycan-dependent protein folding (calnexin cycle), which are closely associated with the Ire1-mediated UPR [25]. Although the pathogenic fungus *C. neoformans* has a evolutionally distinct dolichol-PP-linked Man_9_GlcNAc_2_ *N*-glycans lacking the terminal glucoses residues, this yeast still has a functional UDP-glucose:glycoprotein glucosylatransferase (UGGT), an essential sensor for misfolded glycoproteins in the calnexin cycle, which can be connected to the ERAD pathway [26]. Besides the calnexin cycle sensor UGGT, the presence of the UPR sensor Ire1 and other folding and degradation enzymes were indicated by bioinformatic analysis of *C. neoformans* homologs involved in ER quality control [27]. However, little is known about the UPR signal transduction pathway in *C. neoformans* because the presence of its key major transcription factor, Hac1/Xbp1 ortholog, has not been found. Here, for the first time we identified and characterized the *C. neoformans* UPR pathway, which is composed of an evolutionarily conserved and a distinct signaling component, an ER stress sensor Ire1 and its downstream bZIP transcription factor Hxl1, respectively. We demonstrated that the Ire1-mediated UPR signaling pathway plays essential roles in ER stress response, thermotolerance, cell wall integrity, and virulence via Hxl1-dependent and -independent pathways in *C. neoformans*.

Whereas Ire1 orthologs are evolutionarily well conserved in terms of structure and function in unfolded protein response and adaptation among most eukaryotes, Hac1/Xbp1 orthologs appear to be relatively less conserved. In this study, we showed that the *C. neoformans* Hxl1 is phylogenetically very divergent from the Hac1/Xbp1 transcription factors in the ascomycetous fungi and animals (Figure 2E). As the case of *C. neoformans*, a recent *in silico* analysis of the corn smut fungus *Ustilago maydis* reported the presence of putative homologs for UPR signaling, Um03841 and Um05045, encoding *ScIRE1* and *ScGCN2* homologs, respectively, but did not find any homolog for *ScHAC1* [28]. Furthermore, the plant homolog for Hac1/Xbp1 has not yet been reported [10], [11]. It is notable that as shown in Figure 2D, the BLASTp analysis using the bZIP domain of *C. neoformans* Hxl1 as query identified several hitherto uncharacterized Hxl1 homologs in several basidiomycetous fungi, such as *Tremella fuciformis* bZIP 1 (e-value = 4e-07), *Coprinopsis cinerea* CC1G_02249 (6e-06), *Laccaria bicolor* LACBIDRAFT_293210 (1e-05), and *U. maydis* UM03509.1 (1e-04). Analysis of the roles of these Hxl1 homologs in other basidiomycetous fungi could provide more clues on the relationship of bZIP transcription factors evolved to function in the UPR pathway to cope with ER stress.

Despite that Hxl1 is phylogenetically very divergent from the Hac1/Xbp1 transcription factors in the ascomycetous fungi and animals, the borders of the unconventional intron of *HXL1* mRNA are predicted to form a typical stem-loop structure of intron regions (Figure S2B), which is probably recognized by Ire1 [29]. The conserved nucleotides CAG at both of the intron borders were identified as the most likely splice sites of *HXL1* mRNA (Figure S2C). However, the highly conserved bipartite element, which is important for *HAC1* mRNA splicing *in vivo* and found in the stem-loop of 3′ untranslated region (UTR) of all *HAC1* orthologs identified thus far [30], [31], was not detected in *HXL1*, indicating the presence of a different splicing mechanism of *HXL1* by Ire1. Moreover, the complementary sequences to the *HAC1* introns, involved in the translational inhibition of unspliced *HAC1* mRNA in *S. cerevisiae* [7], was not detected in the 5′ UTR of *HXL1* mRNA, indicating that the regulation of *HXL1* expression at translational level might be different from that of *HAC1* in *S. cerevisiae*. Length of the unconventional intron of *CnHXL1* mRNA (56-nt) is longer than that of mammalian *XBP1* mRNA or some other fungal orthologs (19∼29-nt), but much shorter than that of *S. cerevisiae HAC1* mRNA (252-nt) [12], [13], [29], [31], [32], [33]. In contrast to the intron of *S. cerevisiae HAC1*, the shorter introns of other fungal *HAC1* ortholog and mammalian *XBP*1 mRNAs do not contain the sequences complementary to their 5′ UTR region and thus no translational attenuation as a regulatory means of UPR activation occurs [7], [32]. Since we could not detect the protein product of the unspliced *HXL1* mRNA (Figure S6), it is speculated that the *HXL1* intron may evolve a functionally distinct role in regulation of UPR in *C. neoformans*. Interestingly, the protein product of spliced *HXL1* mRNA was observed only under ER stress condition, but not under normal, unstressed condition regardless of the presence of 20–30% splicing event of *HXL1* mRNA (Figure S6 and Figure 2C). Although its reason is not clear at this point, we speculate that *HXL1* mRNA may partially undergo splicing event, but its protein product is not stably maintained under unstressed condition. This hypothesis is currently under investigation.

We reported that the *C. neoformans* strains lacking Ire1 or Hxl1 display multiple defects not only in ER function but also in cell wall integrity, thermotolerance, and virulence. However, the most notable and significant function of the UPR pathway in *C. neoformans* we discovered in this study is its absolute requirement for survival and proliferation at 37°C and within the mouse host. The ability to grow and survive at physiological body temperature is an essential virulence attribute of human pathogenic microorganisms. Although the precise reasons for the extreme thermosensitivity of the UPR mutants are not clear at this point, one possibility can be deduced from the recent reports of transcriptome analysis of *C. neoformans* during growth at high temperature [21], [34], [35]. These analyses showed that genes involved in stress response, cell wall integrity, filamentation, oxidative metabolism, protein targeting, and fatty acids metabolism are induced during growth at 37°C. We observed that in *C. neoformans*, the expression of *ALG7* and *KAR2* was up-regulated by the Ire1/Hxl1-dependent pathway under heat stress and expression of some other genes involved in *N*-/*O*-glycosylation was also significantly dependent on the presence of Hxl1 during heat stress. Particularly, defects of *N-/O*-glycosylation were shown to cause thermosensitivity, decreased stability and increased misfolding of glycoproteins with various cellular functions [36], [37]. Thus, down-regulation of those genes by loss of the UPR function in *C. neoformans* may cause severe defects in several cellular processes upon heat stress. Besides controlling thermotolerance, the UPR pathway might control other virulence factors of *C. neoformans*. During host infection, *C. neoformans* may activate the secretory pathway for secretion of host degrading enzymes, such as urease, phospholipases, proteinases, as well as transport of polysaccharide-filled vesicles. In this process, the UPR pathway could be induced to enhance the secretion and folding capacity. Therefore, we stress that the Ire1-Hxl1-mediated UPR system plays important roles in ER quality control and is essential during growth and cell survival for cryptococcal disease.

Interestingly, the *ire1* strain expressing the activated *HXL1* ^s^ was not fully recovered for thermotolerance, capsule formation, or virulence compared with the *ire1*+*IRE1* complemented strain (Figure 5, 7, and 8). Furthermore, we suggest that Ire1 and Hxl1 govern the UPR of *C. neoformans* by similar and distinct mechanisms through expression analysis of a few UPR target genes in UPR mutant strains (Figure 6). Similarly, in the nematode *Caenorhabditis elegans*, many canonical UPR-induced genes are regulated by both *ire-1* and *xbp-1* [38]. However, some subsets of constitutive UPR genes, whose expression are not induced by TM but dependent on ER stress transducers, showed a different requirement for either *ire-1* or *xbp-1* [38]. In *Drosophila* and mammalian cells, Ire1 also has an Xbp1-independent role, as part of the regulated Ire1-dependent decay pathway, where it can selectively degrade groups of mRNAs encoding secreted or integral membrane proteins to relieve the burden on the ER [39], [40]. Collectively, we suggest that Ire1 has a broad range of physiological roles in *C. neoformans* in both Hxl1-dependent and -independent manners.

**Figure 10:**
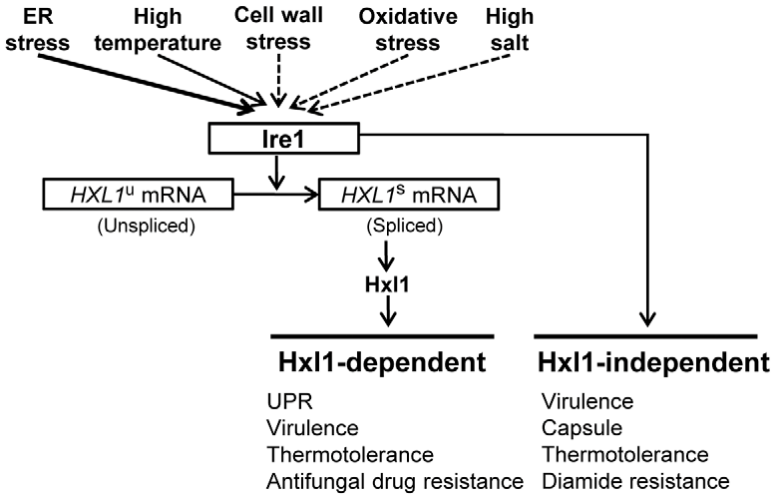
The proposed role of the UPR pathway as core stress response to various stresses in *C. neoformans.* The Ire1-Hxl1-mediated UPR system plays important roles in ER quality control and is essential during growth and cell survival for cryptococcal disease. Notably, Ire1 has a broad range of physiological roles in *C. neoformans* not only in Hxl1-dependent but also Hxl1-independent manners. Solid arrow indicates direct involvement of the UPR pathway (e.g., ER stress) and dotted arrow indicates the direct or indirect activation of the UPR pathway via potential cross-talk with other signaling pathways.

In conclusion, we demonstrated that a unique UPR pathway composed of an evolutionarily conserved Ire1 protein kinase and a novel Hxl1 transcription factor functions not only in ER stress response, but also in controlling cell wall integrity, growth at host physiological temperature, and virulence of the human pathogenic yeast *C. neoformans*. Moreover, the Mpk1 MAPK pathway regulating cell wall integrity was shown to be activated by TM treatment or defects in UPR, supporting the essential role of UPR as a common defense mechanism for both ER and cell wall stress in *C. neoformans*. Based on our data, we propose that the Ire1-Hxl1-mediated UPR serve as a core stress response pathway to various environmental cues in *C. neoformans*, cross-talking with other signaling pathways directly or indirectly (Figure 10). Furthermore, our findings suggest a novel antifungal therapeutic target for specific treatment of *C. neoformans*. Intriguingly, Hxl1 is widely conserved in different serotypes of *C. neoformans* and *C. gattii*. We propose Hxl1 is an ideal target for antifungal drug development, based on its marked divergence from the host Xbp1 transcription factor. In the future, genome-wide comparative expression analysis of *ire1* and *hxl1* mutant strains under several stress conditions, including ER stress and thermal stress, will shed light to further understand and identify UPR target genes and differential regulation of the Hxl1-dependent and -independent Ire1 signaling pathways.

## Materials and Methods

### Ethics statement

The animal studies at Duke University Medical Center were in full compliance with all of the guidelines of the Duke University Medical Center Institutional Animal Care and Use Committee (IACUC) and the United States Animal Welfare Act (Public Law 98–198). The Duke University Medical Center IACUC approved all of the vertebrate studies. The studies were conducted in Division of Laboratory Animal Resources (DLAR) facilities that are accredited by the Association for Assessment and Accreditation of Laboratory Animal Care (AAALAC).

### Strains, media, plasmids, and primers

The *C. neoformans* strains used in this study are listed in Table 1. Yeast strains were maintained and cultured in YPD (1% yeast extract, 2% peptone, 2% glucose) medium. Niger seed medium for melanin production, and agar-based Dulbecco's modified Eagle's medium for capsule production were prepared as described previously [41]. The plasmids and primers used in this study listed in the Table S1. Information on genomic DNA sequences for each gene was obtained from the *C. neoformans* serotype A genome database (Duke university/Broad Institute of Harvard), and the *C. gattii* serotype B genome database (Broad Institute of Harvard/MIT).

### Gene disruption and complementation of *IRE1* and *HXL1*

Gene disruption of *IRE* and *HXL1* was carried out in the *C. neoformans* serotype A H99 (*MAT*α) strain background by using overlap PCR or double joint (DJ)-PCR strategies combined with biolistic transformation as described previously [42], [43] and schemes of their gene disruption were illustrated in Figure S1. For the *IRE1* disruption, the first 5′ and 3′ UTR regions of the *IRE1* gene were PCR-amplified with the primer sets B1644/B1645, B1646/B1647, respectively, by using H99 genomic DNA as a template, and the dominant selectable marker *NAT* (nourseothricin acetyltransferase) was amplified with the M13Fe/M13Re primers from pNATSTM#224. A 3.6 kb *IRE1* disruption cassette was generated by overlap PCR with the primers B1644/B1647 from the combined templates of the first PCR products. The *IRE1* disruption cassettes were precipitated onto 600 µg of gold microcarrier beads (0.6 µm, Bio-Rad) and strain H99 was biolistically transformed. For the *HXL1* gene disruption, 5′ and 3′ UTR regions were amplified with primer sets B1881/B1882 and B1883/B1884 from H99 genomic DNA as template, and the *NAT* markers were amplified with primer sets M13Fe/B1455 and M13Re/B1454 from the vector pNATSTM#229. A *HXL1* gene disruption cassette generated by DJ-PCR was precipitated onto 600 µg of gold microcarrier beads and biolistically transformed into the strain H99 in serotype A. Stable transformants selected on YPD medium containing nourseothricin were initially screened by diagnostic PCR and the correct gene disruptions were further confirmed by Southern blot analysis (Figure S1).

To generate *ire1*+*IRE1* complemented strains, an *IRE1* gene fragment containing its 0.78 kb promoter, 3.52 kb ORF, and 0.53 kb terminator was PCR-amplified using the primers B1969/B1970 including NotI restriction sites from the genomic DNA of strain H99. The 4.8 kb PCR product of the *IRE1* gene was subcloned into pTOP-V2 (Enzynomics), generating a plasmid pTOP-IRE1. Then the *IRE1* gene insert was subcloned into plasmid pJAF12 [41] containing the *NEO* marker (neomycin/G418-resistant marker) to produce pJAF12-IRE1. MluI-digested linearized pJAF12-IRE1 was re-integrated into the native *IRE1* locus of the *ire1* mutants (YSB552 and YSB554) by biolistic transformation, generating the *ire1*+*IRE1* (YSB1000 and YSB1005) strains. To generate *hxl1*+*HXL1* complemented strains, the 1 kb promoter and the 0.6 kb terminator fragments of the *HXL1* gene were PCR-amplified with two primer sets C35/C36 and C37/C38, respectively, from the genomic DNA of strain H99and fused by the second round PCR using primers C35/C38. Then the fusion-PCR product was subcloned into pJAFS1 which is derived from pJAF12 by deleting a SacI site, resulting in a vector pJAFS1-HXL1PT. An unspliced version of the *HXL1* gene (*HXL1* ^u^) and a spliced version of the *HXL1* gene (*HXL1* ^s^) were PCR-amplified using the primers C39/C40 from the cDNAs generated from total RNAs isolated from cells treated without or with TM, respectively. The *HXL1* ^u^ and *HXL1* ^s^ PCR products were then cloned into pJAFS1-HXL1PT, generating pJAFS1-HXL1u and pJAFS1-HXL1s, respectively. The vectors were digested at a unique SacI site of the *HXL1* promoter and reintegrated into the native *HXL1* locus of the *ire1* (YSB552 and YSB554) strain. To generate an *hxl1*+*HXL1* complemented strain, pJAFS1-HXL1u digested with SacI was reintegrated into the native *HXL1* locus of the *hxl1* (YSB723) strain.

### Total RNA preparation, RT-PCR, and qRT-PCR

For UPR induction, cells at OD_600_ (optical density at 600 nm) = 0.15 were grown to mid-logarithmic phase (OD_600_ = 0.5) in YPD at 30°C and then treated with 5 or 8 µg/ml TM, or 20 mM DTT, for 1 or 2 hr. The cell pellets were washed with diethylpyrocarbonate-treated water twice, immediately frozen in liquid nitrogen, and disrupted thoroughly using a mortar and pestle. Total RNAs were extracted using the RNeasy Mini Kit (Qiagen) and subsequently single stranded cDNA was synthesized using a MMLV reverse transcriptase (Invitrogen). Reverse transcriptase-polymerase chain reaction (RT-PCR) was conducted using serially diluted cDNA with Maxime PCR PreMix (i-Taq) (iNtRON Biotechnology). Quantitative real-time PCR (qRT-PCR) was performed using a CFX96 Real-Time PCR detection system (Bio-Rad). Normalized fold expression (2^−ΔΔCT^) was calculated with the CFX manager software using *ACT1* as a reference gene and graphed with Microsoft Excel. All PCR reactions were analyzed in duplicate and all primers used in the study are listed in Table S1.

### Construction of *HXL1* expression vectors for complementation analysis in *S. cerevisiae hac1*

For construction of an expression vector of *HXL1* under the control of the *S. cerevisiae GAL10* promoter, the DNA fragments of unspliced and spliced *HXL1* were amplified with two pairs of primers, C43/C44 and C43/C45, respectively, and subcloned into pRE316 harboring the *GAL1*/*10* promoter, *CEN*, and *URA3*, resulting plasmids pRE316-CnHXL1u and pRE316-CnHXL1s, respectively. For an expression vector of *HXL1* using the *S. cerevisiae HAC1* promoter, the *ScHAC1* promoter and the *ScHAC1* ^u^ fragment were amplified with the primer pairs C46/C47 and C48/C49, and then subcloned into pRE316, generating pRE318-ScHAC1u. The *ScHAC1* ^u^ fragment of pRE318-ScHAC1u was then replaced with the *ScHAC1* ^s^ fragment, which was amplified with the primers C48/C50, or the *HXL1* ^u^, and the *HXL1* ^s^ fragments, generating plasmids pRE318-ScHAC1s, pRE318-CnHXL1u, and pRE318-HXL1s, respectively. *S. cerevisiae* transformants harboring the *S. cerevisiae HAC1* or *C. neoformans HXL1* expression vectors were selected on media lacking uracil and assayed for the sensitivity to TM and DTT. For construction of an *HXL1* integration vector under the control of the *S. cerevisiae HAC1* promoter, the 2.3 kb *ScHAC1* promoter-*HXL1* ^u^ or -*HXL1* ^s^ fragments were obtained from pRE318-CnHXL1u or CnHXL1s, respectively, via treatment of NheI/PvuII and Klenow fragment, and ligated to pTcUR3 [44] treated with BamHI and Klenow fragment, generating pTcU-CnHXL1u and pTcU-CnHXL1s, respectively. These vectors were transformed into wild-type BY4742 and *Schac1* strains after AvrII digestion.

### Drug sensitivity test

Strains grown in YPD medium overnight were spotted onto solid YPD medium containing the indicated concentrations of tunicamycin (TM), dithiothreitol (DTT), Congo red (CR), Calcofluor white (CFW), diamide, and antifungal drugs [azoles (ketoconazole, fluconazole, itraconazole), polyene (amphotericin B), and phenylpyrrole (fludioxonil)], incubated at 30°C, and observed as previously described [24].

### Western blotting analysis of Mpk1 phosphorylation

Total proteins were extracted as described previously [45] and analyzed by Western blotting with phospho-p44/42-MAPK antibody (#4370, Cell Signaling Technology) for Mpk1-P protein and anti-Hog1 antibody (sc-9079, Santa Cruz biotechnology) for the Hog1 protein as a loading control.

### Capsule and melanin assay

Levels of capsule and melanin production were qualitatively and quantitatively measured as described previously [41]. Statistical analysis of relative capsule diameter was performed by using the Bonferroni multiple comparison test.

### Animal studies

Four- to six-week-old female A/Jcr mice (Jackson Laboratory, 18–22 g) were utilized in this study. For infection, strains were cultured in YPD medium overnight at 24°C, washed twice with sterile phosphate buffered saline (PBS), and resuspended in sterile PBS at 2×10^6^ cells per ml. Serially diluted cells were plated onto YPD medium and incubated at 24°C for 72 hr to determine viability. Ten mice per strain (except strains YSB723 and YSB747 for which nine mice each were studied) were anesthetized with pentobarbital (Lundbeck Inc.) and infected via intranasal instillation with 10^5^ cells (in 50 µl). Survival was monitored twice daily, and moribund mice were CO_2_-euthanized. The Kaplan-Meier survival curves were generated by Prism 5.02 (GraphPad Software), and *P* values were evaluated from a Log-rank (Mantel-Cox) test.

To determine fungal burden, the lungs and brains of *C. neoformans*-infected mice (n = 5 for each strain, except n = 4 for strains YSB1000 and YSB762 due to death after pentobarbital treatment) were dissected at day 7. Half organ portions were weighed, transferred to a 15-ml Falcon tube filled with 5 ml PBS, and homogenized for 10 s at 13,600 rpm/min (Power Gen 500, Fisher Scientific). Tissue homogenates were serially diluted, and 200 µl was plated onto a YPD plate. The plates were incubated at 24°C for 48 h to determine CFU per gram of lung or brain. The significance of differences in fungal burden was determined using one-way analysis of variance (ANOVA) and Bonferroni multiple comparison test. For histopathological analysis, half organ samples of lung and brain were fixed in 10% phosphate-buffered formalin (Fisher), and Mucicarmine and hematoxylin and eosin (H&E) staining were performed by the Department of Pathology at Duke University. After slide preparation, each sample was examined thoroughly by microscopy for analysis of *Cryptococcus* colonization (Mucicarmine) and tissue necrosis (H&E). Images were captured using an Olympus Vanox microscope (PhotoPath; Duke University Medical Center).

### General techniques

Protein domain analysis was performed with SMART [46]. Multiple sequence alignments were constructed with CLUTAL W (http://www.genome.jp/tools/clustalw/) and shaded using Boxshade 3.21 (http://www.ch.embnet.org/software/BOX_form.html). Percent identity of proteins was calculated by comparing sequence pairs in relation to the phylogeny reconstructed by the CLUSTAL W method of the Lasergene MegAlign program (DNASTAR Inc.). Searches for the presence of the UPRE and UPRE-like sequences without any mismatches in the promoter region (−1000∼−1) of the putative *C. neoformans* UPR regulated genes were performed with RSA-tools-dna-pattern (http://rsat.ulb.ac.be/rsat/) [47].

## Supporting Information

Figure S1 Disruption of *C. neoformans IRE1* and *HXL1.* (A) Diagram for disruption of the *IRE1* gene in serotype A strain H99 (left panel) and Southern blot analysis (right panel). (B) Diagram for disruption of the *HXL1* gene in strain H99 and Southern blot analysis. Primers used for gene disruption are indicated as bent arrows. The *IRE1* and *HXL1* genes were specifically deleted with the *NAT*-dominant selectable marker in strain H99. The correct gene disruptions were confirmed by Southern blot analysis using genomic DNAs digested with the indicated restriction enzyme.
(TIF)

Figure S2 Structural characteristics of the *HXL1* genes in *C. neoformans* strains of different serotypes. (A) Non-conventional spliced intron sequences of serotype A, D, and B strains. cDNA nucleotide sequences of the *HXL1* genes were aligned from +901 to +1392 of serotype A and D, and +998 to +1334 of serotype B. The unconventional intron sequences are displayed in lowercase with a dotted line, and asterisks represent the stop codon for each of the unspliced and spliced *HXL1* mRNAs, respectively. (B) Secondary structure of the unconventional introns of *C. neoformans HXL1, C. elegans* (AAL60200), and *H. sapiens* (NP_005071) *XBP1* mRNAs. RNA secondary structure prediction was performed with the CLC RNA benchwork 4.0 Demo program (CLC bio). Conserved sequences in splicing junctions are indicated by dotted boxes. (C) Comparison of the unconventional intron region sequences of *HAC1*/*XBP1*. The mRNA sequences surrounding the unconventional introns in *C. neoformans HXL1, Y. lipolytica HAC1*, *C. albicans HAC1*, *T. reesei hac1*, *A. nidulans hacA*, *C. elegans* and *H. sapiens XBP1* are aligned. Flanking sequences are indicated by uppercase, and intron sequences are in lowercase. Asterisks represent the conserved nucleotide sequences among the intron junctions.
(TIF)

Figure S3 Quantification of relative ratio of *HXL1* ^u^ and *HXL1* ^s^ and *KAR2* mRNA levels under normal and UPR-induced conditions. (A) For specific amplification of *HXL1* ^u^ mRNA by qRT-PCR, C79 and C80 primers were designed to bind only to the unconventional (UC) intron region and to the C-terminal exon region, respectively. For amplification of *HXL1* ^s^ mRNA by qRT-PCR, C81 and C82 primers were designed to bind to the exon junctions and to the C-terminal exon region, respectively. (B) The qRT-PCR analysis of *HXL1* ^u^, *HXL1* ^s^, and *KAR2* mRNAs. *C. neoformans* strains were cultivated in YPD with or without TM (8 μg/ml) or DTT (20 mM) as described in Figure 2C. The relative expression levels of *HXL1* ^u^ and *HXL1* ^s^ mRNAs (left panel), and *KAR2* mRNA (right panel) were analyzed by qRT-PCR with primers represented in (A) and listed in Table S1 in duplicate, and normalized to that of *ACT1*. (C) Relative quantification of RT-PCR products of *HXL1* ^u^, *HXL1* ^s^, and *KAR2* mRNAs in Figure 2C. (D) Relative quantification of RT-PCR products of *HXL1* ^u^, *HXL1* ^s^, and *KAR2* mRNAs in Figure 9B. The intensities of *HXL1* ^u^, *HXL1* ^s^, *KAR2*, and *ACT1* were quantified by analyzing the band intensity of corresponding RT-PCR product in the gels with a Quantity One 4.6.2 software (Bio-Rad) in triplicate and calculated as the relative % ratio of *HXL1* ^u^ and *HXL1* ^s^ mRNAs and as the relative fold intensity of *KAR2* to *ACT1*, respectively.
(TIF)

Figure S4 RSA-tools-dna-pattern analysis of putative UPR genes of *C. neoformans* harboring the UPRE and UPRE-like sequences. A set of promoter sequences (-1000∼-1) of putative UPR regulated genes of *C. neoformans* were obtained from the *C. neoformans* genome database and analyzed for the presence of the UPRE (CAGNGTG) and UPRE-like sequences (ATGGTATCAT, TGACGTCA, CCAGC, CGTGTCGG, ACGTGTCG, CGTGTCC, TACGTG, AGTAGGAC, AGGACAAC) by RSA-tools-dna-pattern. Solid bars indicate the exactly matched sequence regions.
(TIF)

Figure S5 Fungal burden assay in the brain. The scatter plot represents CFU (per gram of organ) recovered in the brain from sacrificed animals infected with WT (H99), *ire1* (YSB552), *ire1*+*IRE1* (YSB1000), *hxl1* (YSB723), and *hxl1*+*HXL1* (YSB762) strains.
(TIF)

Figure S6 Translation of *C. neoformans HXL1*. An unspliced *HXL1* expression vector with an N-terminal HA tag under by its own promoter (upper panel) was integrated into WT (H99) and *ire1* strains. Proteins were extracted from WT (H99, lanes 1-4) and *ire1* (lane 5-8) strains incubated in YPD medium treated with (lanes 3-4, lanes 7-8) or without (lanes 1-2, lanes 5-6) TM (5 μg/ml) at 30°C for 2 hr and detected by Western blotting with anti-HA antibody (lower panel).
(TIF)

Table S1 Plasmids and primers used in this study.
(DOC)

Table S2 Identity of overall and the bZIP domain sequences of five *C. neoformans* ORFs to other Hac1/Xbp1 proteins.
(DOC)
