# Supplementary figures and images for: Unique Evolution of the UPR Pathway with a Novel bZIP Transcription Factor, Hxl1, for Controlling Pathogenicity of Cryptococcus neoformans
Source: PLoS Pathog. 2011 Aug 11;7(8):e1002177. doi: 10.1371/journal.ppat.1002177 (PMC3154848; doi:10.1371/journal.ppat.1002177)

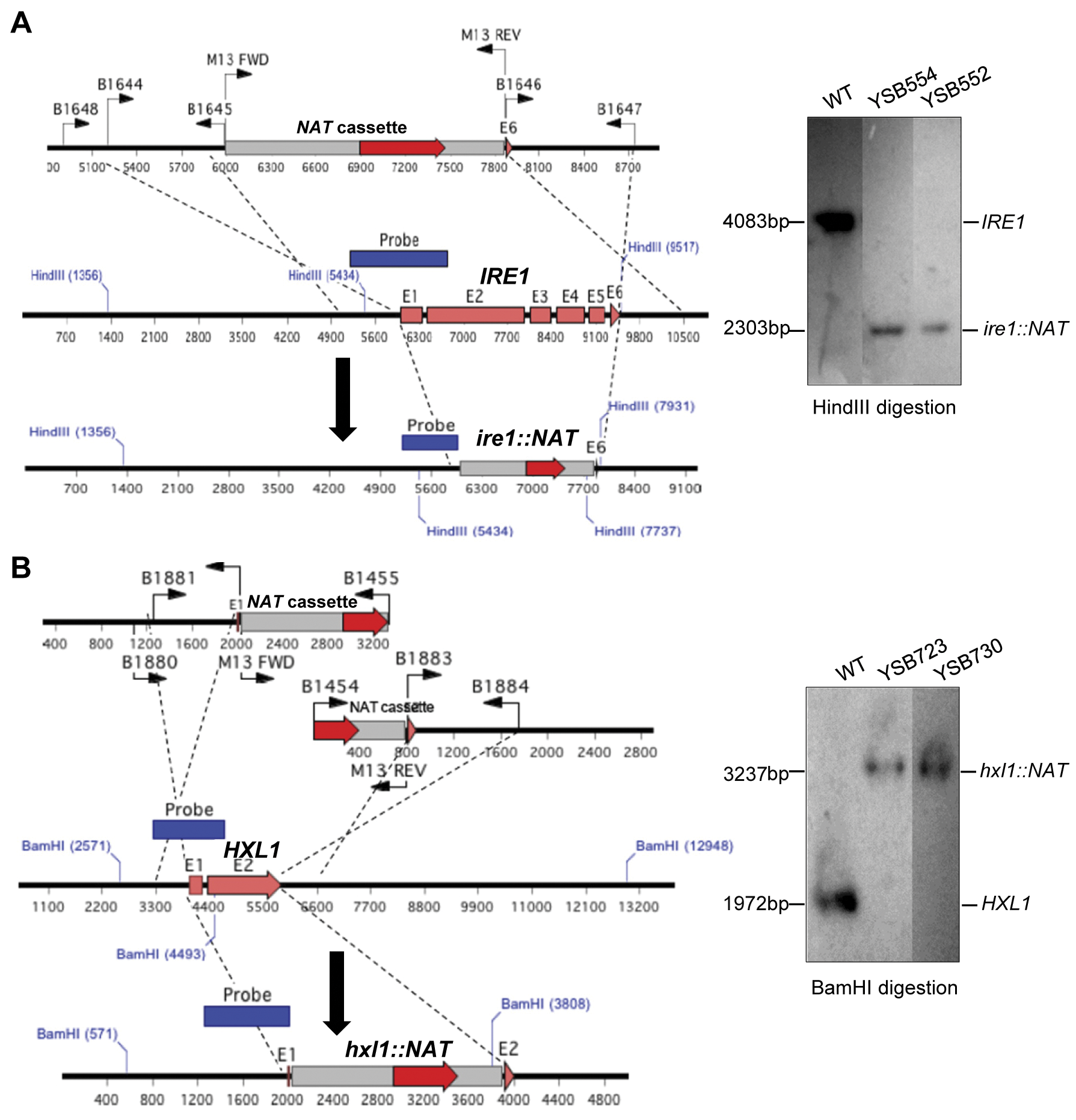

Supplement: Figure S1 — Disruption of C. neoformans IRE1 and HXL1. (A) Diagram for disruption of the IRE1 gene in serotype A strain H99 (left panel) and Southern blot analysis (right panel). (B) Diagram for disruption of the HXL1 gene in strain H99 and Southern blot analysis. Primers used for gene disruption are indicated as bent arrows. The IRE1 and HXL1 genes were specifically deleted with the NAT-dominant selectable marker in strain H99. The correct gene disruptions were confirmed by Southern blot analysis using genomic DNAs digested with the indicated restriction enzyme. (TIF) [file ppat.1002177.s001.tif]

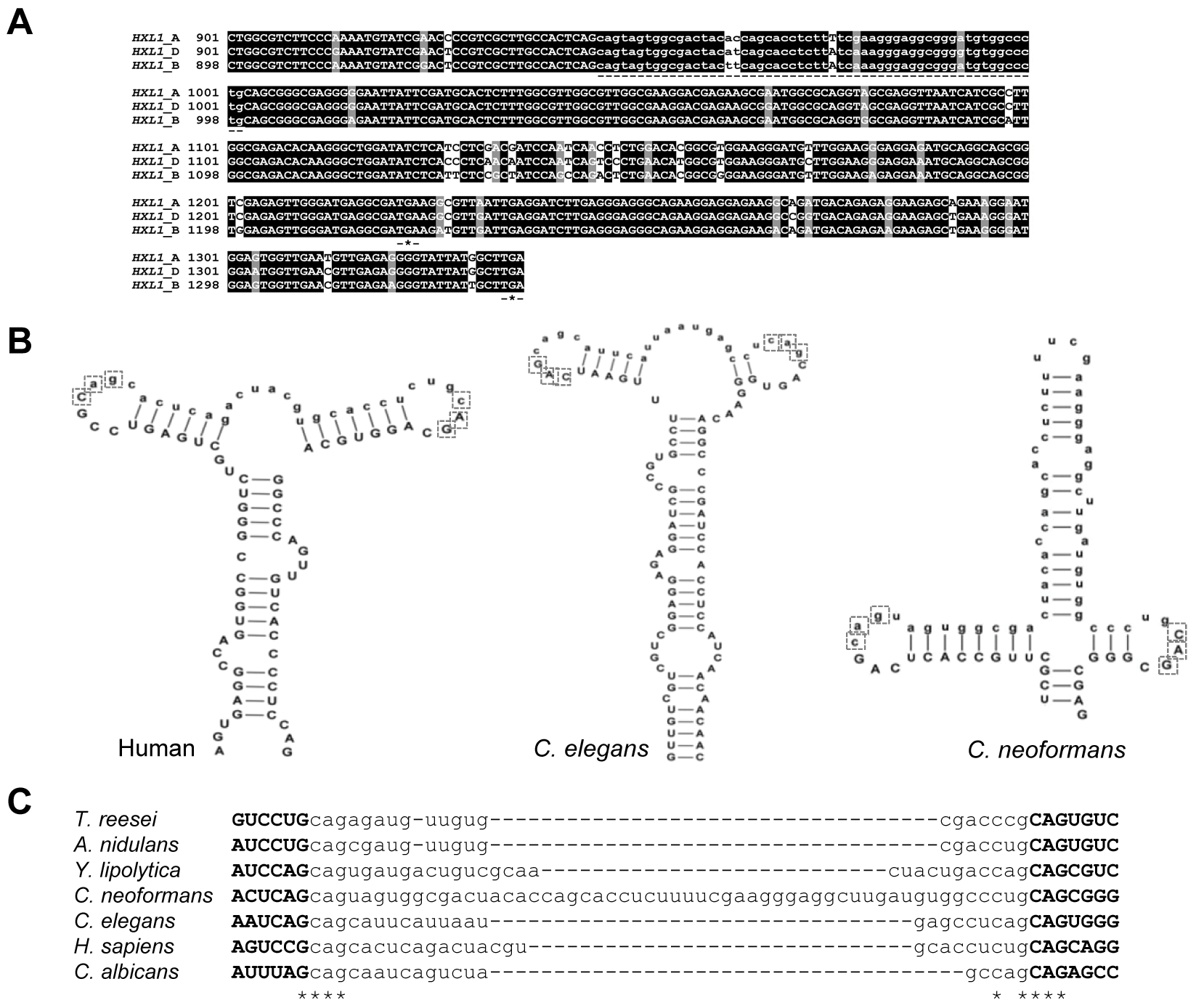

Supplement: Figure S2 — Structural characteristics of the HXL1 genes in C. neoformans strains of different serotypes. (A) Non-conventional spliced intron sequences of serotype A, D, and B strains. cDNA nucleotide sequences of the HXL1 genes were aligned from +901 to +1392 of serotype A and D, and +998 to +1334 of serotype B. The unconventional intron sequences are displayed in lowercase with a dotted line, and asterisks represent the stop codon for each of the unspliced and spliced HXL1 mRNAs, respectively. (B) Secondary structure of the unconventional introns of C. neoformans HXL1, C. elegans (AAL60200), and H. sapiens (NP_005071) XBP1 mRNAs. RNA secondary structure prediction was performed with the CLC RNA benchwork 4.0 Demo program (CLC bio). Conserved sequences in splicing junctions are indicated by dotted boxes. (C) Comparison of the unconventional intron region sequences of HAC1/XBP1. The mRNA sequences surrounding the unconventional introns in C. neoformans HXL1, Y. lipolytica HAC1, C. albicans HAC1, T. reesei hac1, A. nidulans hacA, C. elegans and H. sapiens XBP1 are aligned. Flanking sequences are indicated by uppercase, and intron sequences are in lowercase. Asterisks represent the conserved nucleotide sequences among the intron junctions. (TIF) [file ppat.1002177.s002.tif]

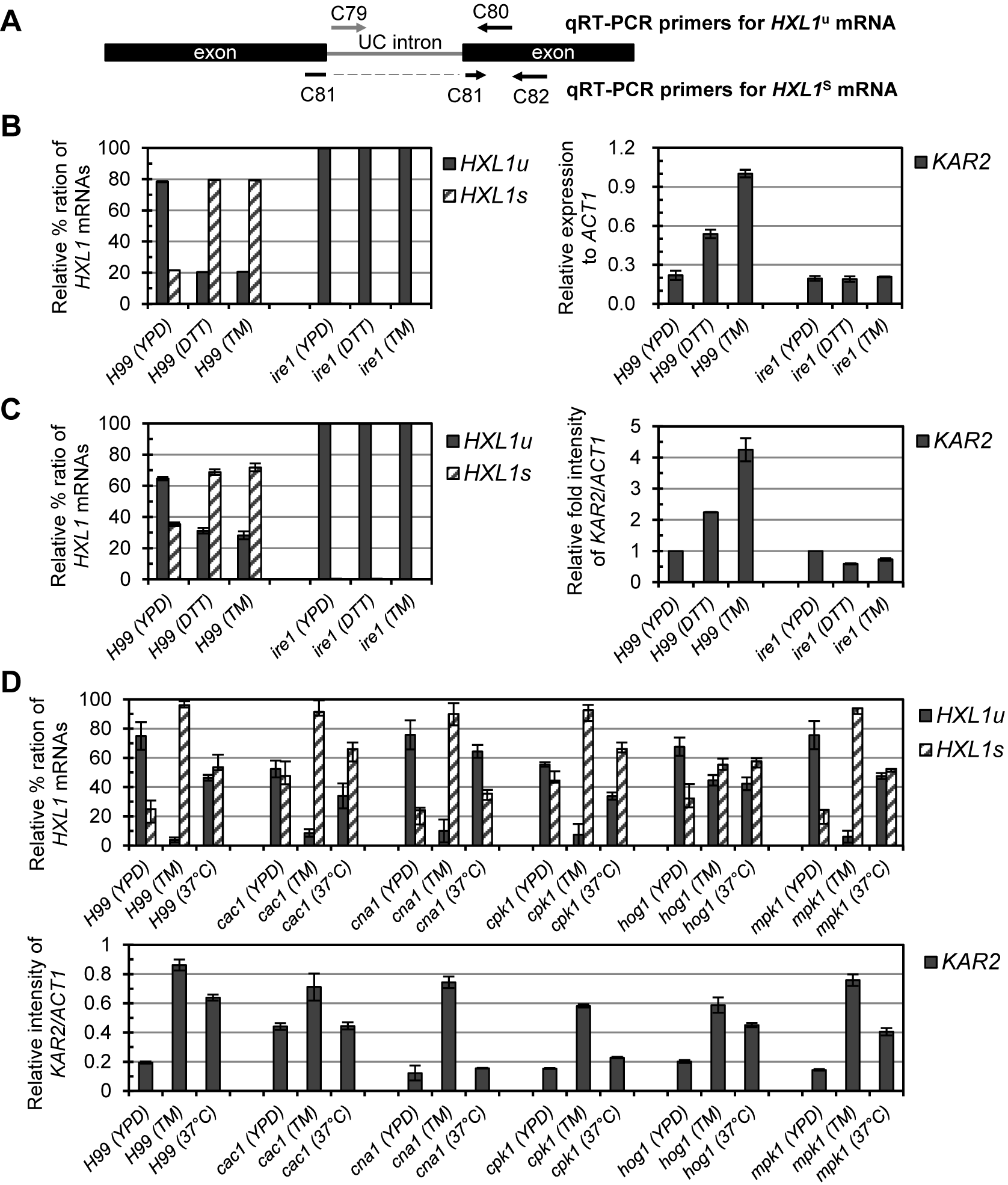

Supplement: Figure S3 — Quantification of relative ratio of HXL1 u and HXL1 s and KAR2 mRNA levels under normal and UPR-induced conditions. (A) For specific amplification of HXL1 u mRNA by qRT-PCR, C79 and C80 primers were designed to bind only to the unconventional (UC) intron region and to the C-terminal exon region, respectively. For amplification of HXL1 s mRNA by qRT-PCR, C81 and C82 primers were designed to bind to the exon junctions and to the C-terminal exon region, respectively. (B) The qRT-PCR analysis of HXL1 u, HXL1 s, and KAR2 mRNAs. C. neoformans strains were cultivated in YPD with or without TM (8 μg/ml) or DTT (20 mM) as described in Figure 2C. The relative expression levels of HXL1 u and HXL1 s mRNAs (left panel), and KAR2 mRNA (right panel) were analyzed by qRT-PCR with primers represented in (A) and listed in Table S1 in duplicate, and normalized to that of ACT1. (C) Relative quantification of RT-PCR products of HXL1 u, HXL1 s, and KAR2 mRNAs in Figure 2C. (D) Relative quantification of RT-PCR products of HXL1 u, HXL1 s, and KAR2 mRNAs in Figure 9B. The intensities of HXL1 u, HXL1 s, KAR2, and ACT1 were quantified by analyzing the band intensity of corresponding RT-PCR product in the gels with a Quantity One 4.6.2 software (Bio-Rad) in triplicate and calculated as the relative % ratio of HXL1 u and HXL1 s mRNAs and as the relative fold intensity of KAR2 to ACT1, respectively. (TIF) [file ppat.1002177.s003.tif]

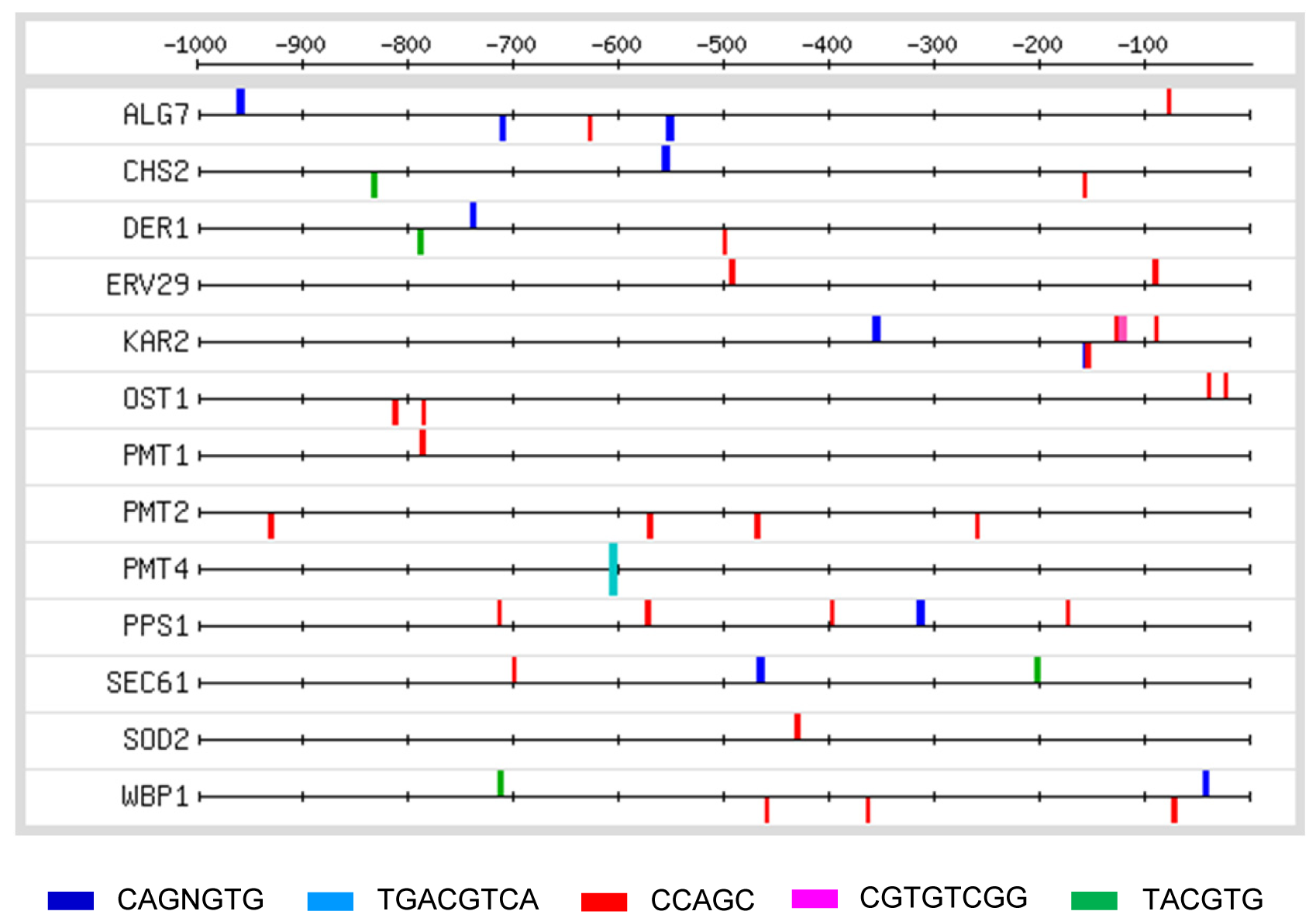

Supplement: Figure S4 — RSA-tools-dna-pattern analysis of putative UPR genes of C. neoformans harboring the UPRE and UPRE-like sequences. A set of promoter sequences (-1000∼-1) of putative UPR regulated genes of C. neoformans were obtained from the C. neoformans genome database and analyzed for the presence of the UPRE (CAGNGTG) and UPRE-like sequences (ATGGTATCAT, TGACGTCA, CCAGC, CGTGTCGG, ACGTGTCG, CGTGTCC, TACGTG, AGTAGGAC, AGGACAAC) by RSA-tools-dna-pattern. Solid bars indicate the exactly matched sequence regions. (TIF) [file ppat.1002177.s004.tif]

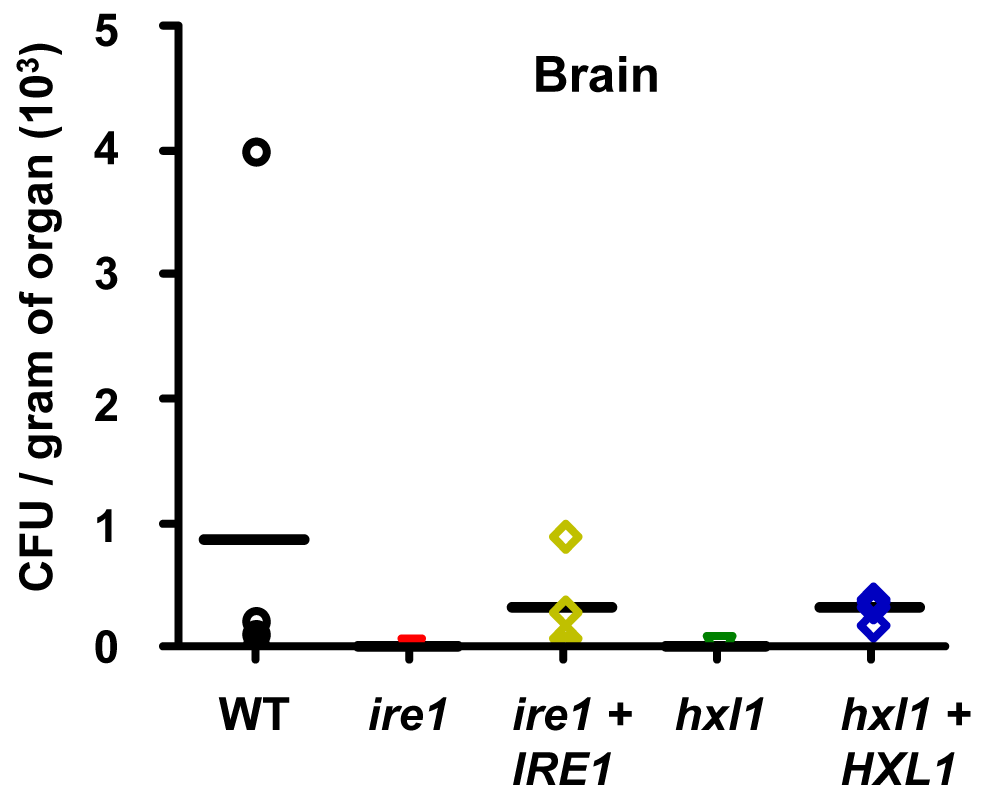

Supplement: Figure S5 — Fungal burden assay in the brain. The scatter plot represents CFU (per gram of organ) recovered in the brain from sacrificed animals infected with WT (H99), ire1 (YSB552), ire1+IRE1 (YSB1000), hxl1 (YSB723), and hxl1+HXL1 (YSB762) strains. (TIF) [file ppat.1002177.s005.tif]

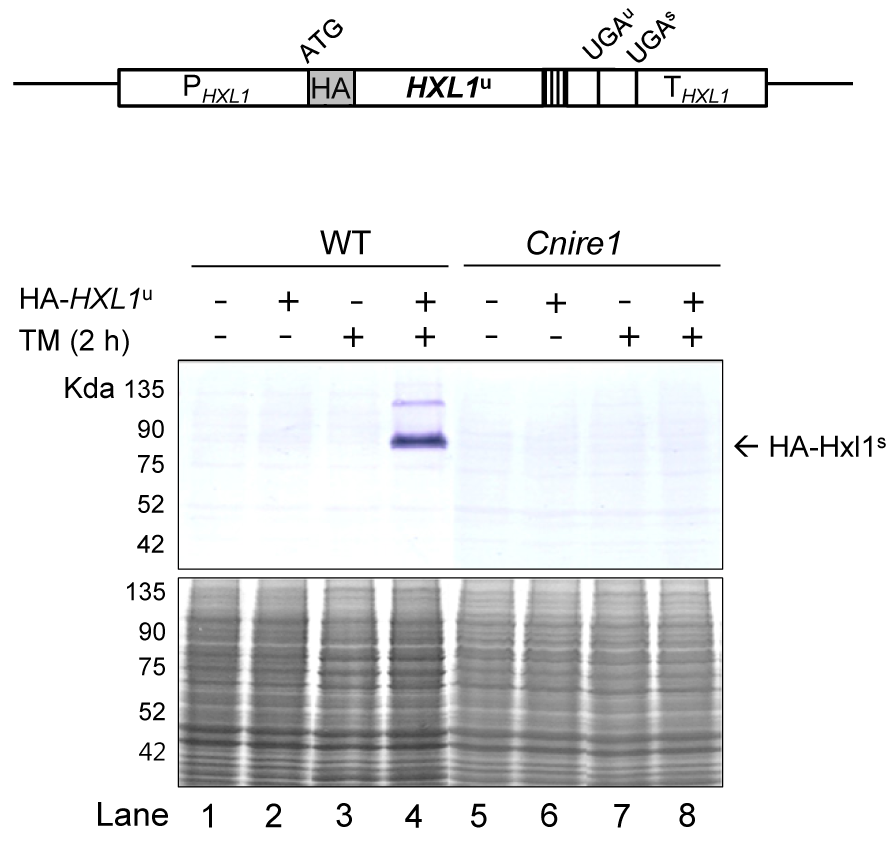

Supplement: Figure S6 — Translation of C. neoformans HXL1. An unspliced HXL1 expression vector with an N-terminal HA tag under by its own promoter (upper panel) was integrated into WT (H99) and ire1 strains. Proteins were extracted from WT (H99, lanes 1-4) and ire1 (lane 5-8) strains incubated in YPD medium treated with (lanes 3-4, lanes 7-8) or without (lanes 1-2, lanes 5-6) TM (5 μg/ml) at 30°C for 2 hr and detected by Western blotting with anti-HA antibody (lower panel). (TIF) [file ppat.1002177.s006.tif]
