# Supplementary material for: Unique Evolution of the UPR Pathway with a Novel bZIP Transcription Factor, Hxl1, for Controlling Pathogenicity of Cryptococcus neoformans
Source: PLoS Pathog. 2011 Aug 11;7(8):e1002177. doi: 10.1371/journal.ppat.1002177 (PMC3154848; doi:10.1371/journal.ppat.1002177)
Supplement: Table S1 — Plasmids and primers used in this study. (DOC) [file ppat.1002177.s007.doc]

**Table S1**. Plasmids and primers used in this study

| **Plasmid** | **Description** | **Reference** |
| --- | --- | --- |
| For *C. neoformans* |  |  |
| pNAT-STM#224 | NAT-resistant marker vector for *IRE1* disruption |  |
| pNAT-STM#229 | NAT-resistant marker vector for *HXL1* disruption |  |
| pJAF12 | *NEO*-resistant marker vector |  |
| pTOP-IRE1 | *IRE1*-containing subcloning vector | This study |
| pJAF12-IRE1 | *IRE1*-containing *NEO*^r^ vector | This study |
| pJAFS1 | Modified pJAF12 vector | This study |
| pJAFS1-HXL1PT | *NEO*^r^ vector 1 containing the promoter and terminator of *HXL1* | This study |
| pJAFS1-HXL1u | *NEO*^r^ vector containing unspliced *HXL1*^u^ containing | This study |
| pJAFS1-HXL1s | *NEO*^r^ vector containing spliced *HXL1*^s^ | This study |
| For *S. cerevisiae* |  | This study |
| pRE316 | p*ScGAL1/10*-*URA3*-*CEN* vector | (Cheon, unpublished) |
| pRE316-CnHXL1u | *pScGAL10*-*CnHXL1*^u^-Flag-*URA3*-*CEN* | This study |
| pRE316-CnHXL1s | p*ScGAL10*-*CnHXL1*^s^-Flag-*URA3*-*CEN* | This study |
| pRE318-ScHAC1u | p*ScHAC1*-*ScHAC1*^u^-Flag-*URA3*-*CEN* | This study |
| pRE318-ScHAC1s | p*ScHAC1*-*ScHAC1*^s^-Flag-*URA3*-*CEN* | This study |
| pRE318-CnHXL1u | p*ScHAC1*-*CnHXL1*^u^-Flag-*URA3*-*CEN* | This study |
| pRE318-CnHXL1s | p*ScHAC1*-*CnHXL1*^s^-Flag-*URA3*-*CEN* | This study |
| pTcU-CnHXL1u | p*ScHAC1*-*CnHXL1*^u^-Flag-tc-*URA3-tc* | This study |
| pTcU-CnHXL1s | p*ScHAC1*-*CnHXL1*^s^-Flag-tc-*URA3-tc* | This study |
| **Primer** | **Sequence (5’ to 3’)^a^** | **Purpose** |
| B1644 | GCCCCATCATCATAATCAC | Disruption primer for *IRE1* |
| B1645 | **GCTCACTGGCCGTCGTTTTAC**ACTATGTGTCCATCTGAGGC | " |
| B1646 | **CATGGTCATAGCTGTTTCCTG**AGTGAGTTGAGGGAGGAAAG | " |
| B1647 | GAAGAAGAGCGTCAAGAAGG | " |
| B1648 | AGGAATACGAGGTTTATCGG | Diagnostic primer for *IRE1* |
| B1683 | AGCATTAGGGGTGTAGGTG | Probe primer for *IRE1* |
| B1881 | GTTTGAGGCTGGTAAAAAGG | Disruption primer for *HXL1* |
| B1882 | GCTCACTGGCCGTCGTTTTACATGGGGAATGAAAGCGTG | " |
| B1883 | CATGGTCATAGCTGTTTCCTGAAGGGGCGAGAGTAGTTCAG | " |
| B1884 | GACTGTAAAGGAGGGCATAAG | " |
| B1880 | AACTCTTCTCAGCCTTCGG | Diagnostic primer for *HXL1* |
| B1885 | CGTTCTCCGTCTTGATAGC | Probe primer for *HXL1* |
| M13Fe | GTAAAACGACGGCCAGTGAGC | Primers for disruption marker |
| M13Re | CAGGAAACAGCTATGACCATG | Primers for disruption marker |
| B1969 | CGCGCGGCCGCGCACAGGATTACTTTTGGGTGATG | *IRE1* complementation |
| B1970 | CGCGCGGCCGCAGTTGGAAAAGGAGCGTCC | " |
| B1454 | AAGGTGTTCCCCGACGACGAATCG | Double joint PCR primer |
| B1455 | AACTCCGTCGCGAGCCCCATCAAC | Double joint PCR primer |
| C01 | ATACAGCCAGTGTCCTTCCC | RT-PCR primer for CNAG_03976.2 of H99 (RT-PCR1) |
| C02 | ATGGATATCAGTTGCGGGAT | " |
| C03 | AGAGATTGAGCTCCTCCG | RT-PCR primer for CNAG_03976.2 of H99 (RT-PCR2) |
| C04 | TTACCAGACACTGACACC | " |
| C05 | ATGTCCGCGATCGATTAC | RT-PCR primer for CNAG_07560.2 of H99 (RT-PCR1) |
| C06 | CCTTCTTCTCAGCAGCAGTC | " |
| C07 | TTAACGGCTGCGTCAATC | RT-PCR primer for CNAG_07560.2 of H99 (RT-PCR2) |
| C08 | TCTTCTTCTATCGACCCG | " |
| C09 | TTTGGCAATGACGACTCAAG | RT-PCR primer for CNAG_07940.2 of H99 (RT-PCR1) |
| C10 | CCTGTAACGCTCTGTTCTCC | " |
| C11 | TCATGGGCTGTTGATTCC | RT-PCR primer for CNAG_07940.2 of H99 (RT-PCR2) |
| C12 | TAAAGGAAGGTTCCGGTG | " |
| C13 | AGTGCACTGATGGCGTCA | RT-PCR primer for CNAG_00871.2 of H99 (RT-PCR1) |
| C14 | AAAGCATAGACAACGGCG | " |
| C15 | CACTCGGCACCGTTATGT | RT-PCR primer for CNAG_00871.2 of H99 (RT-PCR2) |
| C16 | TGGCAAATGCGTAGCTTC | " |
| C17 | ATGGCTACCGCTGTCGCT | RT-PCR primer for CNAG_06134.2 of H99 (RT-PCR1) |
| C18 | TGATTCGCGGTTACGGAT | " |
| C19 | CACTCCATTCCTTTCTGC | RT-PCR primer for CNAG_06134.2 of H99 (RT-PCR2) |
| C20 | CGTAACTCCACTGTGTCC | " |
| C21 | CGTAACTCTACTGTCTCC | RT-PCR primer for CNM1380 of JEC21 and JEC20 |
| C22 | ATTTCCTCCCTTCCAAGC | " |
| C23 | CACTCCATCCCCTTCAGC | RT-PCR primer for CNBG_4842.2 of R265 and WM276 |
| C24 | CGTAACTCTATCGTCTCG | " |
| C25 | TGCAGAAGATGGCGTTGC | RT-PCR primer for *IRE1* of all serotypes |
| C26 | ACACTCCCGCCTTTATAC | " |
| C27 | TCGATGCCAATGGTATCC | RT-PCR primer for CNAG_06443.2 |
| C28 | TCATGGCTGAAAGGCATC | " |
| C29 | CACTCACCGATCTGTTTC | RT-PCR primer for CNAG_00072.2 |
| C30 | ATTTGCTTGGCAGAGTCC | " |
| C31 | ACCCAAGGTCCTGTTTAC | RT-PCR primer for 06240.2 |
| C32 | ATCGTGCTCAGGAGTCTC | " |
| C33 | AGCCTTCTCTCCTTGGTC | RT-PCR primer for *ACT1* |
| C34 | ACGATTGAGGGACCAGAC | " |
| C35 | GTCGTTAACCTTCGGAGGCTTTTACAC | *HXL1*complementation |
| C36 | **CTCGAGCATATGGCTAGC**TGCGAGGATGGGAATAGG | " |
| C37 | **GCTAGCCATATGCTCGAG**GGAAGAAACAAAATAACCAAC | " |
| C38 | CACGGTACCAATATATCATGCCCTCCCG | " |
| C39 | GCAAGCTAGCATGGCTACCGCTGTCGCT | *HXL1* complementation and *HXL1* cloning for serotype A and D |
| C40 | CCACTCGAGTCAAGCCATAATACCCCTCT | " |
| C41 | ATGGCTACCGTTGTCGCT | *HXL1* cloning for serotype B |
| C42 | TCAAGCAATAATACCCTTCTC | " |
| C43 | CGGAATTCATGGCTACCGCTGTCGCT | *HXL1* complementation for *S. cerevisiae* |
| C44 | ATCTATCGATTGTCGCCTCATCCCAACTCT | " |
| C45 | ATCTATCGATTGAGCCATAATACCCCTCTC | " |
| C46 | TATGCTAGCCGGCAGACAATGCAGAAG | " |
| C47 | CGAGAATTCAGTGGCGGTTGTTGTCGT | " |
| C48 | CGGAATTCATGGAAATGACTGATTTTGAAC | " |
| C49 | ATCTATCGATTGCTGTAGTTTCCTGGTCATCG | " |
| C50 | ATCTATCGATTGTGAAGTGATGAAGAAATCATTC | " |
| C51 | CTTCCAGCCTTCTCTCCTTG | qRT primer for *ACT1* (CNAG_00483.2) |
| C52 | AGAGGTCCTTCCTGATGTCG | " |
| C53 | CTCTGAGGACGACAAGGACA | qRT primer for *KAR2* (CNAG_06443.2) |
| C54 | AGCTCAGAAAGCTGCTCCTC | " |
| C55 | TATCACTGCGCCATATCTCC | qRT primer for *DER1* (CNAG_06365.2) |
| C56 | AGCTGCTCTGACACCTCCTT | " |
| C57 | ATCATGTCCTCCGACTCCTC | qRT primer for *SEC61* (CNAG_06998.2) |
| C58 | TGATACCGAGCTCCATCAAG | " |
| C59 | GTCGCTATCCAGGGACATTT | qRT primer for *ALG*7(CNAG_06901.2) |
| C60 | GTCCAAAGAGTTGAGGGCAT | " |
| C61 | GGAGAAGGCTGTAGGTGGAA | qRT primer for *WBP1* (CNAG_04743.2) |
| C62 | TGGCATTATCTCTGGTCTGC | " |
| C63 | GCATTGGTTACGGTCTCCTT | qRT primer for *ERV29* (CNAG_04651.2) |
| C64 | GCAAAGAGTCGGAGAGAACC | " |
| C65 | CGCCCATTCTGCCTACTATT | qRT primer for *OST1* (CNAG_04715.2) |
| C66 | TTGGGTGAGAATGCTACCAG | " |
| C67 | CTCGTACGCTGTGCTTGATT | qRT primer for *PMT1* (CNAG_06834.2) |
| C68 | CCGAGCTCATAAACCCATCT | " |
| C69 | CTCTCCCATACGCCTACGAT | qRT primer for *SOD2* (CNAG_04388.2) |
| C70 | TGCCTTCTGGAGAGACTCCT | " |
| C71 | GCGTGCTTGATTTGACTGAT | qRT primer for *PPS1* (CNAG_05639.2) |
| C72 | CTCCAACACGACAATGAACC | " |
| C73 | CCGAGTCTCCTCTCGAAATC | qRT primer for *PMT2* (CNAG_04763.2) |
| C74 | GAAGACTTCCCACGGGTAAA | " |
| C75 | ACAATGCGGGTCTTACTTCC | qRT primer for *PMT4* (CNAG_00996.2) |
| C76 | TGCTGACCCTCATTACGTGT | " |
| C77 | GATCGTATTCTCGCGTTTGA | qRT primer for *CTS2* (CNAG_03326.2) |
| C78 | TTGAGCCATCTTCTTCGTTG | " |
| C79 | TAGTGGCGACTACACCAGCA | qRT primer for *HXL1*^U^ |
| C80 | CAACGCCAAAGAGTGCATC | " |
| C81 | CTTGCCACTCAGCAGCGG | qRT primer for *HXL1*^S^ |
| C82 | CAAGGCGATGATTAACCTCG | " |

^a^The underlined sequences in the primers are restriction enzyme sites for subsequent subcloning and the bold sequences are complementary sequences for fusion-PCR.

**References**

1. Kim MS, et al. (2009) An efficient gene-disruption method in *Cryptococcus neoformans* by double-joint PCR with NAT-split markers. *Biochem Biophys Res Commun* 390:983-988.

2. Bahn YS, et al. (2004) Adenylyl cyclase-associated protein Aca1 regulates virulence and differentiation of *Cryptococcus neoformans* via the cyclic AMP-protein kinase A cascade. *Eukaryot Cell* 3:1476-1491.
