# Supplementary material for: Unique Evolution of the UPR Pathway with a Novel bZIP Transcription Factor, Hxl1, for Controlling Pathogenicity of Cryptococcus neoformans
Source: PLoS Pathog. 2011 Aug 11;7(8):e1002177. doi: 10.1371/journal.ppat.1002177 (PMC3154848; doi:10.1371/journal.ppat.1002177)
Supplement: Table S2 — Identity of overall and the bZIP domain sequences of five C. neoformans ORFs to other Hac1/Xbp1 proteins. (DOC) [file ppat.1002177.s008.doc]

**Table S2.** Identity of overall and the bZIP domain sequences of five *C. neoformans* ORFs to other Hac1/Xbp1 proteins.

| Percent identity of overall sequence  (amino acid number of unspliced/spliced forms)^1^ | | | | | Percent identity of bZIP domain sequence  ( bZIP region) | | | | |
| --- | --- | --- | --- | --- | --- | --- | --- | --- | --- |
| Cn | Sc (230 aa/  238 aa) | Af (433 aa/  342 aa) | Ce (287 aa/  285 aa) | Hs (261 aa/  376 aa) | Cn  (bZIP domain) | Sc (37-95 aa) | Af (78-142 aa) | Ce (59-123 aa) | Hs (48-132 aa) |
| CNAG_00871.2 (499 aa) | 13.5/19.4 | 11.4/15.4 | 16.7/13.1 | 20/16.1 | (42-122 aa) | 28.8 | 20 | 24.6 | 33.8 |
| CNAG_06134.2 (407 aa) | 17.3/16.9 | 15.1/14.8 | 11.2/14 | 15.9/15.2 | (60-125 aa) | 28.8 | 20 | 23.1 | 29.2 |
| CNAG_07560.2 (403 aa) | 11.7/8.7 | 12.3/11.5 | 14.7/10.2 | 15.3/10.2 | (122-187 aa) | 16.9 | 18.5 | 30.8 | 32.3 |
| CNAG_07940.2 (342 aa) | 13.3/14.2 | 11.3/16.7 | 12.6/12 | 17/13.2 | (85-146 aa) | 22 | 23 | 27.9 | 31.1 |
| CNAG_03976.2 (508 aa) | 7.4/12.7 | 6.2/7.6 | 10.1/3.2 | 12.6/10.1 | (267-331 aa) | 23.7 | 21.9 | 26.2 | 26.2 |

^1^Sc, *S. cerevisiae* Hac1 (accession number of unspliced form, BAA05513/accession number of spliced form, NP_116622); Af, *A. fumigates* HacA (XP_748727/ACJ61678); Ce, *C. elegans* (AAL60200/AAL60201); Hs, *H. sapiens* (NP_005071/NP_001073007)
